# Tablet- and Group-Based Multicomponent Cognitive Stimulation for Older Adults With Mild Cognitive Impairment: Single-Group Pilot Study and Protocol for Randomized Controlled Trial

**DOI:** 10.2196/64465

**Published:** 2025-02-21

**Authors:** Khanitin Jornkokgoud, Pattrawadee Makmee, Peera Wongupparaj, Alessandro Grecucci

**Affiliations:** 1 College of Research Methodology and Cognitive Science Burapha University Chon Buri Thailand; 2 Department of Psychology and Cognitive Science (DiPSCo) University of Trento Rovereto Italy; 3 Department of Research and Applied Psychology Faculty of Education Burapha University Chon Buri Thailand; 4 Department of Psychology Faculty of Humanities and Social Sciences Burapha University Chon Buri Thailand; 5 Centre for Medical Sciences (CISMed) University of Trento Trento Italy

**Keywords:** computerized cognitive stimulation, multisensory integration, cognitive decline, aging, electroencephalography, randomized controlled trial, RCT, protocol, cognitive stimulation, mild cognitive impairment, cognitive, cognition, cognitive simulation therapy, CST, MCI, tablet, effectiveness, pilot study, neuropsychological tests, behavioral, emotional

## Abstract

**Background:**

Cognitive stimulation therapy is a group-based psychological treatment for people with dementia as well as those with mild cognitive impairment (MCI) and is shown to improve both cognition and quality of life. Previous studies have indicated the potential to benefit from the use of technological devices in group interventions.

**Objective:**

The pilot study aimed to assess the effectiveness of a tablet- and group-based multicomponent cognitive stimulation therapy (MCST) for enhancing cognitive functions among older adults with MCI. The following study aims to report the protocol for a trial evaluating whether the MCST program is affecting individuals with MCI.

**Methods:**

In the first study, 30 individuals with MCI participated in 10 sessions of the tablet- and group-based MCST group. A subsequent protocol study will compare tablet-based MCST, tablet-based cognitive stimulation therapy, and control groups among 93 individuals with MCI. All participants will be recruited from older adults living in semiurban communities. Intervention groups will be facilitated by trained therapists, nurses, or psychologists. The study will be assessed by a pre- and posttest evaluation, including computer-based neuropsychological tests and electroencephalography assessment. The effects of several indicators, such as cognitive functions, behavioral, and emotional, will be analyzed as being indexed by their neurophysiological data.

**Results:**

The pilot study showed significant cognitive improvement (*P*<.001), reduced depression (*P*=.002), and decreased state anxiety (*P*=.001) post intervention. Quality of life remained unchanged (*P*=.18). The randomized controlled trial study was funded in March 2023. Enrolling began in August 2023 and was completed in December 2023. The data analysis was started, and the results are expected to be published by mid- to late-2025.

**Conclusions:**

The study is the first tablet-group–based MCST for older adults with MCI in middle-income countries. It will provide deeper insight into participants’ neuropsychological data, thus identifying specific processes underlying physiologically measured positive outcomes. Furthermore, the project will deliver solid and integrative results to mental health professionals in terms of knowledge and guidance for implementing the tablet- and group-based MCST in people with MCI.

**Trial Registration:**

Thai Clinical Trials Registry TCTR20230829004; https://tinyurl.com/3wuaue3e

**International Registered Report Identifier (IRRID):**

DERR1-10.2196/64465

## Introduction

Mild cognitive impairment (MCI) is the stage between normal aging and dementia, including probable Alzheimer disease, characterized by noticeable cognitive deficits that do not impair daily functioning significantly [1-3]. The cognitive domains affected by MCI include learning and memory, language, visuospatial abilities, executive functions, and psychomotor skills. Notably, evident impairment in any of these domains is sufficient for an MCI diagnosis [4]. Furthermore, particular deficits in emotions such as anger, sadness, and fear have been observed in individuals with MCI, with anxiety being more prevalent in clinical samples than in community-based ones [5,6]. In addition, subjective cognitive decline has been linked to anxiety symptoms, independently increasing the risk of MCI or dementia [7]. Depression is also associated with early cognitive impairment, but patients with depression typically do not exhibit the memory deficits observed in MCI or dementia [8,9].

Group-based cognitive stimulation therapy (CST) has been shown to improve both cognitive functions and quality of life in older adults with dementia or MCI [10,11]. The guiding principles of CST were adapted to create 15 fundamental principles of individual-centered CST, including mental stimulation, reminiscence, learning and communication stimulation, and a person-centered approach. The program includes various activity sessions encompassing different areas such as life history, current affairs, creative tasks, games, and cognitive challenges [12,13]. The belief that continuous participation in various mental activities improves cognitive and social functioning underpins cognitive stimulation treatments [14]. Furthermore, Silva and colleagues [15] discovered that in MCI or mild dementia, a cognitive stimulation program could account for the improved cognition response observed. These data reveal that the lower the cognitive damage, the better the neuroplastic capacity and ability to learn, and the greater the potential to induce neurogenesis. Therefore, cognitive interventions should be implemented at the earliest stages of cognitive impairment [15].

Recent studies on tablet-based intervention with cognitive stimulation have focused on the feasibility, acceptability, and cognitive and psychosocial effects of the computerized cognitive stimulation (CCS) and computerized cognitive engagement programs. Both treatments were efficient and acceptable, allowing patients with MCI to improve in several aspects of their cognitive and psychosocial functioning. Still, the effect sizes on cognition, such as free recall and the Trail Making Test part A, were moderate, favoring the CCS group [16]. A further study explored whether CCS induced differential effects in older adults with MCI according to the degree of white matter hyperintensities, were separated into no-to-little and moderate-to-severe groups. Following the session, both groups improved on numerous cognitive tests but not in mood and psychosocial features except for motivation [17]. Previous research suggested that the effectiveness of tablet-based CST on cognition, emotion, or psychosocial outcomes should be investigated [17]. Furthermore, the increased computerization of intervention programs is a step toward treatment uniformity. Computer-based intervention methods are cost-effective, noninvasive, and simple to execute, requiring little human and financial resources [18]. In particular, tablet-based CST should also be further examined in middle-income or developing countries since the expected findings will close the gaps and expand the generalizability of the theories and therapeutic technologies.

In addition, extant research shows that CST is effective in enhancing cognitive functions in people with mild dementia investigated by using the resting-state functional magnetic resonance imaging technique [19] that provided solid evidence of enhancement in the neuronal networks in terms of structures and functionalities by using a group CST. Nonetheless, the previous research came with methodology limitations regarding the small sample sizes and comparison between groups. Furthermore, there is a lack of studies to show the effect of changes in neurobiological features in older adults with MCI. Thus, this study aims to monitor the effectiveness of tablet-based CST using neuroimaging techniques in people with MCI.

The underlying neurobiological factors contributing to MCI involve changes in neurotransmitters, including the noradrenergic, serotonergic, and dopaminergic systems [20]. Notably, the cholinergic system, particularly acetylcholine, plays a crucial role in cognitive function, with its decline correlating with cognitive impairment. Electroencephalography (EEG) measures have revealed alterations in brain activity patterns among individuals with MCI, including increased beta-two power and notable changes in the theta, alpha, and delta frequencies [21]. Atrophy of the hippocampus and the medial temporal lobe regions, along with hypometabolism in specific brain areas, are indicative of MCI [22]. Specifically, electrophysiological recordings and event-related potentials (ERPs) provide valuable insights into cognitive functioning. P300 latency delays have been observed in people living with MCI, and abnormalities in N400 and P600 components are associated with a higher risk of developing Alzheimer disease [23-26]. These EEG and ERP components could potentially serve as biomarkers for monitoring brain change in people with MCI.

Accordingly, the first study aims to evaluate the effects of the tablet- and group-based multicomponent cognitive stimulation therapy (MCST) program on cognition and emotions in older adults with MCI. The program draws on CST principles and multisensory stimulation techniques to create tailored activity sessions that target cognitive function and emotions. This study hypothesizes that the intervention positively affects cognition and emotions in older adults with MCI after the intervention.

The following study aims to assess how a tablet-based MCST program, with or without multisensory integration (MSI), affects cognitive functions, emotions, and quality of life in older adults with MCI, comparing experimental and control groups designed as parallel groups. Changes in EEG and ERPs will be examined before and after the intervention. The cognitive domains targeted as primary outcomes for improvement include learning and memory, language, visuospatial abilities, executive functions, and psychomotor skills as well as emotions. Specific brain locations are associated with these domains, and the study aims to enhance the functioning of these regions to counteract the cognitive decline observed in MCI. The protocol study hypothesizes that the intervention will improve cognitive functions, emotions, and behaviors in older adults with MCI, outperforming an active comparator and control group. Expected EEG and ERP changes may reveal brain activity patterns tied to cognitive improvements, focusing on frequency bands and ERPs.

## Methods

### Overview

The pilot and protocol for a randomized controlled trial (RCT) intervention study have been approved by the Burapha University institutional review board (IRB4-191/2566). The study was conducted according to the Guideline for Good Clinical Practice and the Declaration of Helsinki [27]. Following the gold standard in research on intervention effectiveness—the CONSORT (Consolidated Standards of Reporting Trials; checklist provided in Multimedia Appendix 1), the RCT study design, including the proximal and distal outcomes, has been preregistered in the Thai Clinical Trials Registry (reference TCTR20230829004).

This project consisted of 2 studies. The first study involved a pilot of the MCST intervention and effective assessment of cognitive functions in older adults, which began in August 2023. The next study is the RCT, with recruitment starting in September 2023. The intervention and data collection were done over 14 weeks.

### Study 1: Pilot Study

#### Study Design

This study investigates the efficacy of the tablet- and group-based MCST program, using a 1-group pretest-posttest design. Participants were randomly assigned to 3 subgroups, and dependent variables were measured both before and after the implementation of the program.

#### Participants

In total, 30 older adults participated as volunteers in the experiment. Participants ranged from 60 to 75 (mean 66.14, SD 4.72 years) years of age with normal or corrected normal vision, no color blindness or weakness, and no history of mental illness or neuropathy. Thai native speakers were capable of reading and comprehending writing. All participants were assessed using the Thai version of the Montreal Cognitive Assessment (MoCA-T) to assess cognitive impairment [28], the subjective memory complaint scale to assess complaints of defective memory [29], the Chula Index Scale and Activities of Daily Living to assess normal activities of daily living [30], and the Clinical Dementia Rating Scale to assess the absence of dementia [31]. These assessments are established screening tools as criteria for detecting MCI [32].

Participant characteristics show that among them, MCI was presented (MoCA-T scores between 17 and 24), 20% (6/30) were male and 80% (24/30) were female. Participants’ occupations varied as 43% (13/30) were farmers, 10% (3/30) were freelancers, 10% (3/30) were sellers, 13% (4/30) were retired government officers, and 23% (7/30) were unemployed. Regarding health conditions, 23% (7/30) of participants had diabetes, while 40% (12/30) reported having hypertension. An additional 20% (6/30) reported other diseases. In terms of vision, 57% (17/30) of participants wore glasses and 43% (13/30) did not wear glasses.

#### Intervention

The MCST interventional program was adapted from group CST for MCI with 10 sessions and once a week over a period of 10 weeks [10]. The intervention is presented in the RCT study and shown in Table 1.

**Table 1:** Cognitive stimulation therapy sessions adapted to Thai culture.

Session	Theme	Activities
1	Physical activity	Finger exercise and using a touch screen and learning to navigate the tablet
2	Music and sound	Stimulating auditory and visual senses using old music and sounds from daily life
3	Childhood	Pictures of singers, actors, or celebrities from the past
4	Food and cooking	Different kinds of foods, recipes, and methods of cooking; being creative
5	Travel	Current affairs, places and sounds, well-known destinations and talking about the hometown
6	Occupations	People’s jobs, using word games and word association
7	Sports	Sporting events, matching the picture to the word, senses, and sounds
8	Shopping	Using money, prices, and calculation
9	Household	Categorizing objects
10	Team games	Number games

#### Assessments

The results of the effectiveness of the tablet-based group intervention were evaluated. At the pre-and postintervention assessment, participants were asked to examine the MoCA-T, Thai geriatric depression scale (TGDS), State-Trait Anxiety Inventory (STAI)–state, and Older People’s Quality of Life Questionnaire (OPQoL)-Brief (refer to Outcome Measures section for further details).

#### Data Analysis

Because the sample size in this study was not large enough to permit the assumption of normality on the study variables, the nonparametric test was used. The Wilcoxon signed-rank test was used to examine the effects of the tablet- and group-based MCST on cognition, emotions, and quality of life. The rank-biserial correlation coefficient (r_B_) was considered as effect size and is interpreted the same as the r coefficient, such as trivial (<0.10), small (0.10), moderate (0.30), and large (0.50) [33]. The JASP (Jeffreys’s Amazing Statistics Program; Version 0.16.2.0; University of Amsterdam), an open-source statistics program, was used for paired samples and effect size analysis [34].

### Study 2: RCT

#### Overview

The RCT aims to assess the effects of the tablet-group–based MCST program on cognitive functions and emotions and quality of life based on the differences between experimental groups with and without MSI compared with a control group, and the changes observed in EEG and ERPs during the pre- and posttest interventions. The study hypothesizes that the intervention affects cognitive functions positively in older adults with MCI after the intervention compared with an active comparator and a control group. Furthermore, it is hypothesized that the intervention improves emotion and behavior in older adults with MCI after the intervention compared with an active comparator and control group. Changes in brain waves are also expected to reveal brain activity patterns associated with cognitive improvement, focusing on frequency bands and ERPs.

#### Trial Design

This research uses an RCT design with an experimental group, an active comparator group, and a control group. Participants are randomly assigned to 1 of the 3 study conditions. Each participant is randomly allocated 1:1:1 to an intervention or control condition. The study is designed as a randomized, controlled trial with 3 parallel groups. Randomization will be performed by Random Allocation Software (Isfahan University of Medical Sciences) [35].

#### Participants

##### Inclusion Criteria

Participants eligible for inclusion must meet the following criteria: (1) language proficiency: participants must be native speakers of the Thai language [32]; (2) age range: the study focuses on individuals aged 60 years or older; (3) objective memory performance: inclusion is based on objective memory impairment assessments [32] using well-established evaluation tools, including the MoCA-T and clinical dementia rating [36]; (4) subjective memory complaints [32]; (5) handedness: participants must be right-handed, as confirmed by the Edinburgh Handedness Inventory; (6) color vision: normal color vision is a requirement, evaluated using the Ishihara Plate Test; (7) health status: individuals with no chronic illnesses, deafness, blindness, or an diagnosed neurological disorder are eligible to participate; (8) cognitive status: eligible participants should not exhibit signs of dementia [32]; (9) reading ability: prospective participants are expected to be capable of reading and comprehending written material; and (10) computer proficiency: participants should be able to use computer devices effectively for the study activities.

##### Exclusion Criteria

The following criteria will lead to exclusion from participation: (1) a diagnosis of dementia: individuals diagnosed with dementia after participating in the study, irrespective of severity, will not be included in the study; (2) systemic illness: participants with concurrent systemic illnesses that could potentially confound the study outcomes will be excluded; and (3) refusal to participate: individuals who decline to participate or express their unwillingness to engage in the study will not be considered for inclusion.

##### Sample Size

The sample size in the study was estimated using the G*Power program [37]. The required sample size considering an α=.05, a Cohen *d*=0.50 (medium magnitude for the effect size) [17,38], and power=0.95, is 26 or 27 participants in each condition, that is, a total of around 80 participants. Assuming a loss of 15% [17,38,39], in the event, the final sample consists of 93 participants, approximately 31 participants in each condition.

##### Recruitment

Older adults with MCI will be recruited via communities such as clubs for older adults and elderly schools in the semiurban area of Chonburi province, Thailand. Announcements regarding the study will be disseminated through radio and social media platforms (Line Messenger [Line Corporation], Facebook [Meta], and so on). Individuals who express interest will receive information and undergo a screening test. If they meet the inclusion criteria, they will be enrolled in this study.

#### Interventions and Control Group

##### Experiment Intervention

**Figure 1:**
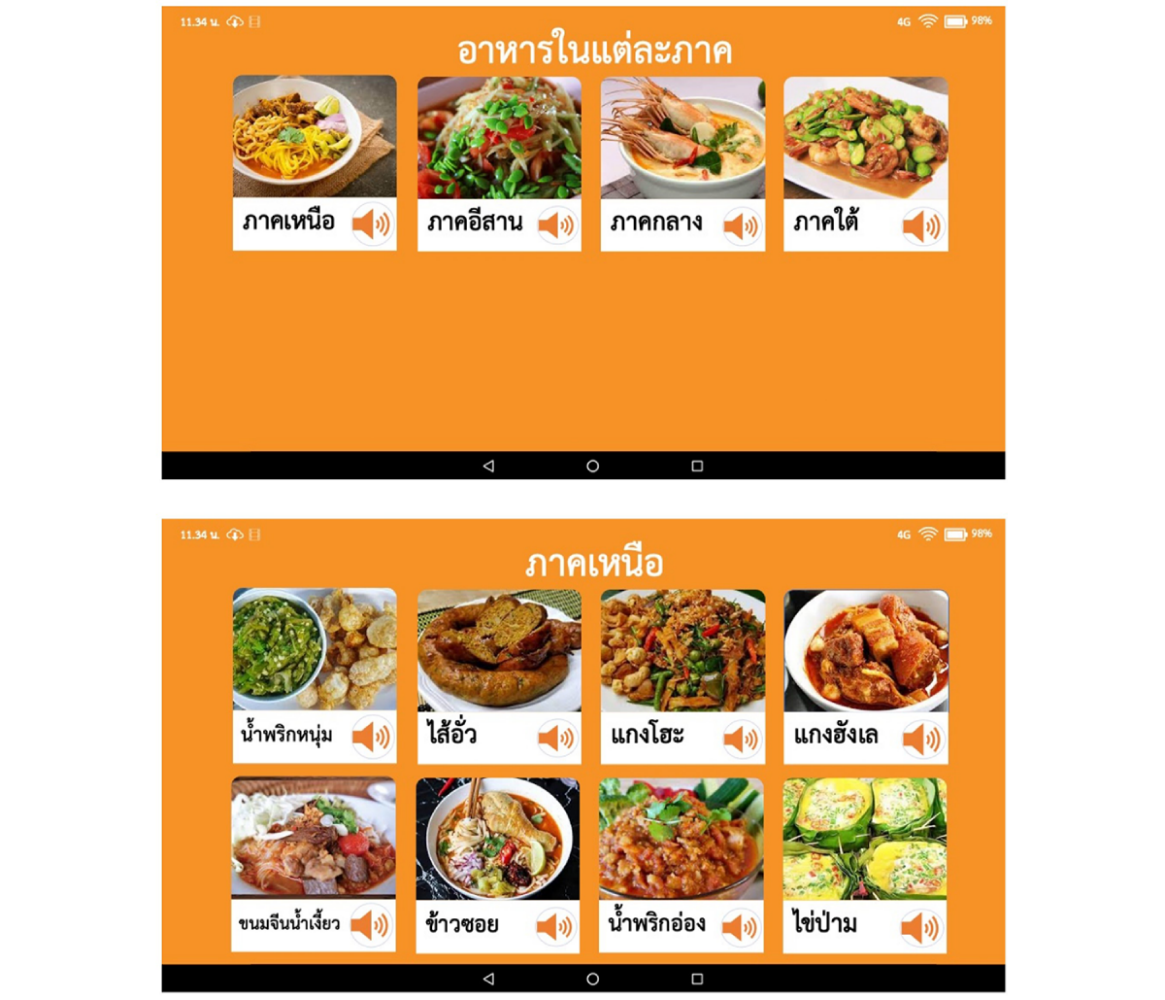
Screenshots of the computerized multicomponent cognitive stimulation app (screenshots present kinds of foods in different regions of Thailand).

The interventional program was adapted from group CST for MCI with 10 sessions [10], the group CST principle [11,13,40], and adapting CST to other cultures [12,38]. Specifically, the program also adopted audiovisual temporal discrimination training to improve MSI [41], which was included in sessions as shown in Table 1 and Figure 1. Participants will be asked to use a 10-inch tablet with an Android operating system (Google) that has the computerized multicomponent cognitive stimulation app for each session installed so that they can participate in all activities. Intervention groups will be facilitated by trained therapists, nurses, or psychologists. Further details of the MCST intervention and the manual can be found on the website [42].

##### Active Comparator

The comparator group is similar to the MCST intervention group but excludes MSI training, receiving only tablet-group–based CST. Each session will be like a structured session in the MCST intervention group.

##### Control Group

The participants in this group will not receive the treatment. They will be measured once at the start and then a second time 2 months later.

#### Data Collection

The study will take place at the Center of Excellence in Cognitive Science, which is part of the College of Research Methodology and Cognitive Science at Burapha University. Demographic and clinical information about age, gender, education, marital status, occupation, diseases, and physical issues will be self-reported at baseline. The results of the effectiveness of the tablet-based group intervention will be evaluated. The outcomes will be evaluated using measures of cognitive functions and brain waves, including EEG and ERP techniques that are recorded while participants perform the tasks.

At the beginning of the EEG and ERP assessment, participants will be asked to wear the neuro handset to correct the EEG resting-state data which will be measured during eyes-closed and eyes-open sessions at 5 minutes. Afterward, the EEG will be recorded while participants are performing the computerized neuropsychological tasks.

Cognitive functions and MSI tasks will use computer-based tasks to access neuropsychological data using PsychoPy software (Open Science Tools Ltd). Individuals will participate in a different sequence when dealing with order effects and time-related factors, changing the order by using counterbalancing. Furthermore, for each task, participants will rest for around 2 minutes before beginning the next task.

Electroencephalogram recordings on the Emotiv EPOC Flex saline and 10-20 layout 32-channel system will be recorded. The reference electrodes are located at the common-mode sensor (left side) and driven-right-leg (right side) sensor. All signals will be filtered automatically with a high-pass filter of 0.2 Hz and a low-pass filter of 45 Hz using a digital fifth-order sync filter and a sampling rate of 128 Hz, and the electrode impedance will be kept to at least 80% using EMOTIV PRO software to monitor EEG quality before recording and to collect the data [43].

Furthermore, after enrollment, the participants will be informed about the experimentation schedule, and the preconditions of the experiment such as proper sleep, and avoiding drinks like coffee and alcohol. Participants are required to shampoo their hair but avoid applying gel or lotion. Before starting the experiment, the researcher will confirm the participant’s preparation before the experiment [44].

### Outcomes Measures

#### Primary Outcomes

##### Learning, Memory, EEG, and ERP Components

The first running requires participants to learn and recall word pairs. The examination contains a variety of 12 pairs of words, such as related words, unrelated words, and name-word pairings. Participants are told to memorize unrelated word pairings (eg, hospital and lawyer). To test later, they are asked to recall those pairs of words. The total time for the test is approximately 10 minutes. A significant change in the recall numbers, EEG and ERP parameters (*P*<.05) is expected.

##### Executive Function Scores, EEG, and ERP Components

A standardized form of the Wisconsin Card Sorting Test, Berg Card Sorting Test will be used with the card sorting task using a 64-card deck. Participants are told to press the 1, 2, 3, or 4 keys that they believe match the card at the bottom of the screen. The cards’ figures differ in color, number, and shape. The classification rule changes every 5 cards. Participants can complete the Wisconsin Card Sorting Test-64 within 10-15 minutes. The scoring includes percentage errors (%), perseverative responses (%), perseverative errors (%), nonperseverative errors (%), conceptual level responses (%), categories completed, trials to complete the first category, failure to maintain set, and learning to learn [45]. A statistically significant change in the EEG and ERP parameters (*P*<.05) is expected.

##### Language Scores, EEG, and ERP Components

The verbal fluency test is a short test of verbal functioning. It typically consists of 2 tasks, that is, category and letter fluency. Participants are given 1 minute to produce as many unique words as possible that are within a semantic category (category fluency: animal and fruit) and start with a given letter (letter fluency: words beginning with the letter Koh and letter Aoh in Thai). The participant’s score in each task is the number of unique correct words [46]. For example, participants answered for animals such as dogs, cats, and monkeys, scoring 3 points for the category. The total time for the test is approximately 5 minutes. A statistically significant increase in fluency scores and EEG and ERP parameters (*P*<.05) is expected.

##### Visuospatial, EEG, and ERP Components

In the Corsi block-tapping task, participants were instructed to tap the blocks in the same serial order as presented (2 trials per sequence length ranging from 2 to 9 blocks). Participants receive 2 trials per level, starting from 2 to 9 lengths. Measures are the span (number in the longest correct sequence with the possible number from 2 to 9), the score (number of correctly reproduced sequences with the potential number from 2 to 9), and the product (span×score) [47]. The total time for the test is approximately 2-5 minutes. On the mental rotation task, participants are asked whether 2 objects rotated relative to one another (geometrical forms) are identical or mirror images [48,49]. The task consists of 69 stimuli, and each correct answer is worth 1 point, making a total of 69 points. Participants can complete the test within 5-10 minutes. A statistically significant increase in all scores, EEG, and ERP parameters (*P*<.05) is expected.

##### Psychomotor, EEG, and ERP Components

In total, 2 reaction time tasks will be used, namely, the Deary-Liewald reaction time task and the number’s reaction time box. In the simple reaction time test participants had to press a button or key in response to a single stimulus. The choice reaction time has 4 stimuli, and participants are asked to press the button corresponding to the correct response. The interstimulus interval ranged between 1 and 3 seconds and will be randomized within these boundaries. The task will record each trial’s response time and the interstimulus interval for measuring [50]. The total time for the test is approximately 3-5 minutes. A statistically significant decrease in response time and total error, EEG, and ERP parameters (*P*<.05) is expected.

##### MSI, EEG, and ERP Components

The audiovisual task consisted of three conditions—2 control conditions (1 beep and 1 flash; 2 beeps and 2 flashes) and the illusion condition (2 beeps and 1 flash; 1 beep and 2 flashes). The auditory and visual stimuli will be presented simultaneously in the control conditions. The visual flash will be delivered simultaneously with the first auditory beep in the illusion condition. In each condition, the stimulus onset asynchronies used are between 150 and 300 milliseconds [51]. For a total of 80 stimuli, participants take approximately 2-3 minutes. Response time and the total error will be assessed. A statistically significant decrease in response time and total error and EEG and ERP parameters (*P*<.05) is expected.

##### The MoCA-T

The MoCA-T version 8 is the most frequently used screening test, and it was produced as a quick screening tool for MCI and dementia in its early phases. The visuospatial (5 points), naming (3 points), attention (6 points), language (3 points), abstract (2 points), memory (5 points), and orientation (6 points) abilities are assessed on this examination. When an assessment was completed, all scores were totaled out of a possible overall of 30. Higher scores indicated more significant cognitive function. The internal consistency of the MoCA-T was excellent (Cronbach α=0.91) [28]. It is expected to see a statistically significant increase (*P*<.05) in global cognition.

##### The TGDS-15

The TGDS-15 is a structured self-report scale that assesses depression symptoms over the previous week. The scale contains 15 items, with responses chosen as agreeing or disagreeing. The scale has a cutoff point for depression, which is 0-5 for no depression, 6-10 for suggestive of depression, and 11-15 for depression [52,53]. The Cronbach α coefficient of The TGDS-15 was 0.83, which showed high internal consistency [53]. It is expected to see a statistically significant decrease (*P*<.05) in depressive symptoms.

##### The STAI

On the State Anxiety Inventory Form Y-I scale, the STAI-state has the 20-item Thai version and has been validated by Thapinta [54]. Participants are asked how often, during the last 2 weeks, they have been bothered by each of the 30 questions of generalized anxiety disorder. Response options are “not at all,” “for several days,” “more than half the time,” and “nearly every day,” scored as 0, 1, 2, and 3, respectively. Therefore, the scores range from 0 to 80, with scores of ≥20, ≥40, and ≥60 representing mild, moderate, and severe anxiety symptom levels, respectively. The Cronbach α coefficient of the Thai STAI-state was 0.90, which showed high internal consistency [54]. A statistically significant decrease (*P*<.05) in the state anxiety symptom is expected.

#### Secondary Outcomes

The OPQoL-Brief in the Thai version [55] consists of 13 questions that are scored strongly agree=1, agree=2, disagree=4, and strongly disagree=5. The items are added together to get a total OPQoL-Brief score, and then positive items are reverse coded, such that higher scores imply better QoL. The entire sum score varies from 13 to 65. The Cronbach α coefficient of OPQoL-Brief was 0.94, showing that the first and the second questionnaire responses with a 2-week interval were highly stable [55]. A statistically significant increase (*P*<.05) in the OPQoL-Brief score is expected.

### Data Analysis

As part of demographic and behavioral data, the means and SDs will be calculated to explain the general description such as age, gender, education, MoCA-T, TGDS-15, STAI-state, and OPQoL-Brief. Cognitive function scores of the 3 groups will be compared using 1-way analysis of covariance (ANCOVA) to examine differences in posttest scores of various neuropsychological tests controlling for pretest scores. The test involves computing an *F*-ratio large enough to indicate significant mean differences. Furthermore, ANCOVA is customary to measure the effect size with partial ω^2^. In addition, post hoc analyses were performed using the Bonferroni Test.

In total, 4 EEG frequency bands’ relative powers will be analyzed, and the latency and amplitude of ERP components will also be assessed. Furthermore, the EEG data collected will be cleaned and manipulated using the EEGLAB toolbox in the MATLAB environment. One-way ANCOVA will be applied to examine differences in the EEG and ERP posttest of various components among intervention groups (MCST, CST, and control) controlling for pretest scores.

### Ethical Considerations

The study was approved by the Burapha University institutional review board (approval IRB4-191/2566; issue date: August 11, 2023; valid until date: August 11, 2024). It was also registered with the Thai Clinical Trials Registry (dated August 29, 2023; TCTR20230829004). All participants provided informed consent. All data will be anonymized. The participants were provided travel compensation (US $33 per participant).

## Results

### Pilot Study

The participants showed significantly improved global cognition as measured by the MoCA-T (*P*<.001), depression as measured by the TGDS (*P*=.002), and state anxiety as measured by the STAI-state (*P*<.001) but not in the quality of life as measured by the OPQoL-Brief (*P*=.18). Furthermore, effect sizes among significant measures were large (r_B_: 0.85-1.00; Table 2).

**Table 2:** Assessment results for older adults with mild cognitive impairment who completed both pre- and posttests.

Measure	Pretest (n=30), mean (SD)	Posttest (n=30), mean (SD)	Wilcoxon Signed Ranks Test			Effect sizes^a^ (95% CI)
			*Z* value	*P* value		
MoCA-T^b^	19.50 (2.13)	23.13 (2.71)	4.70	<.001	1.00 (1.00 to 1.00)	
TGDS^c^	3.53 (1.89)	2.40 (1.10)	2.99	.002	.85 (0.61 to 0.95)	
STAI^d^-state	43.37 (5.40)	37.30 (5.87)	3.96	.001	.87 (0.73 to 0.94)	
OPQoL^e^-Brief	52.47 (10.83)	56.00 (5.97)	1.33	.18	.28 (–0.13 to 0.61)	

^a^The rank-biserial correlation coefficient (r_B_).

^b^MoCA-T: Thai version of the Montreal Cognitive Assessment.

^c^TGDS: Thai geriatric depression scale.

^d^STAI: State-Trait Anxiety Inventory.

^e^OPQoL: Older People’s Quality of Life Questionnaire.

### The RCT

**Figure 2:**
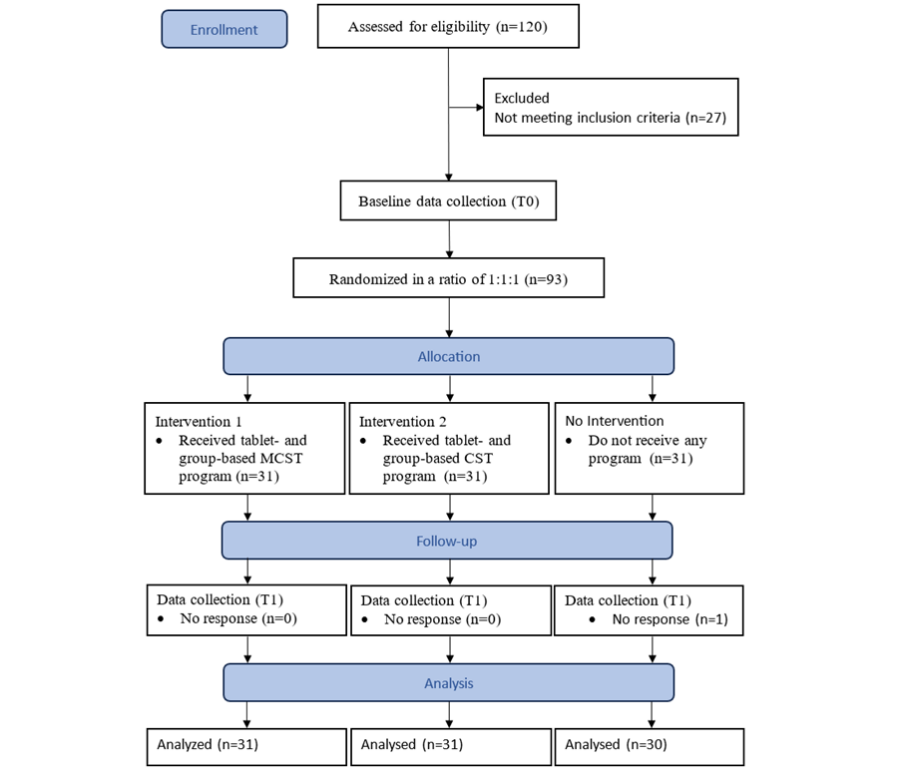
CONSORT (Consolidated Standards of Reporting Trials) flow diagram. CST: cognitive stimulation therapy; MCST: multicomponent cognitive stimulation therapy; T0: baseline for pretreatment assessment (ie, week 0); T1: the week immediately after the intervention (ie, week 10).

In the RCT, the project secured funding in March 2023, started in April 2023, and received recommendations and approval from the Burapha University institutional review board in August 2023. Baseline data collection was conducted in September 2023, and the postintervention data collection started in December 2023. From baseline, we have data from 93 older adults with MCI. Results from the study will be available in mid- to late-2025 at the earliest and will be published in peer-reviewed international and national journals, as well as presented at relevant conferences. An overview of the trial flow is presented in Figure 2.

## Discussion

### Findings From the Pilot Study

The pilot study determined the cognitive function, emotions, and quality of life among older adults with MCI in Thailand. Such improvement was attributed to the tablet- and group-based MCST for 45 minutes per session and once a week over a period of 10 weeks aimed at improving their cognition, emotions, and quality of life. Findings showed that older people with MCI showed improvement in global cognitive function, as proven by scores on MoCA-T. Furthermore, this study also indicated a reduction in depression and anxiety, as demonstrated by scores on TGDS and STAI-state. However, the intervention cannot enhance the quality of life among participants. These findings support that the tablet- and group-based MCST program effectively improves global cognitive function and emotions in older adults with MCI.

The study findings align with previous research regarding CCS using tablet-PCs and social interactions among older adults with MCI [16,17]. A recent study employed the KODRO app (Altera-Group) on Android tablets, incorporating functions like cognitive games, communication tools, and entertainment features to engage various cognitive domains in participants with MCI. The investigation demonstrates that CCS using tablet-PCs enhances cognitive function [16], consistent with meta-analytical findings indicating cognitive training’s positive impact on global cognitive function, memory, and working memory in older patients with MCI [56]. Further evaluation of specific cognitive domains will involve neuropsychological assessments and EEG in older adults with MCI.

In terms of emotion, depression and anxiety are common in people with MCI [57-59]. Fascinatingly, emotional symptoms, such as depression and anxiety, decreased following the session. Contrary to the previous study [16], there was unchanged postintervention. Nonetheless, other findings found that not only cognitive functions but also depression and anxiety were improved in people with MCI after they participated in the computerized cognitive training program [60]. The MCST program’s social interaction through discussions and games may explain the findings, aligning with research showing that social engagement enhances cognitive interventions. This discovery aligns with previous research, indicating that social interaction has the potential to modify the outcome of cognitive intervention programs [61].

In contrast to our hypotheses, this study had no significant effect on the participants’ quality of life. Perhaps because all of our participants were aware of their diagnosis and expressed low satisfaction with their cognition and physical well-being, no change in their quality of life was seen after postintervention. It has been shown that patients with MCI who are aware of their diagnosis or have high memory complaints have a poorer quality of life than those who are oblivious to their diagnosis or have low memory complaints [62,63]. However, previous studies showed that reminiscence therapy improved cognitive performance and quality of life, supporting CST’s use for cognitive decline [64]. Cognitive stimulation benefits daily activities in MCI [10], while another study reported enhanced social, physical, and mental health [65]. Londos et al [66] noted the quality of life gains from goal-oriented rehabilitation, though self and mood subscales showed no significant change. This suggests that enhancing the quality of life in patients with MCI who are aware of their diagnosis may require more targeted interventions.

### Principal Results for the RCT Study

The protocol for the RCT study introduces a comprehensive cognitive intervention program designed to address cognitive functions in older adults with MCI. The proposed program is tailored to target both cognitive domains and MSI. The study hypothesizes that the intervention has a positive effect on cognitive performance in older adults with MCI compared with an active comparator and a control group. In addition, it is anticipated that changes in brainwaves, with a focus on frequency bands and ERPs, are also expected to reveal patterns of brain activity associated with cognitive improvement. This study’s contribution lies in its novel approach to combining CST and MSI training to address those cognitive deficits associated with MCI. The inclusion of tablet-based interventions and interactive sessions adds a technological dimension to traditional cognitive interventions, thereby potentially enhancing engagement and outcomes.

The study draws upon established principles from CST and MSI techniques. The cognitive stimulation program is designed based on group-based CST and multisensory stimulation approaches. CST has been shown to improve cognitive function and quality of life in individuals with dementia or MCI [10,11]. Furthermore, the group-based cognitive stimulation program, which involves social support from friends and group activities, is critical for mental health and adherence to health-promoting behaviors. It can reduce feelings of loneliness and isolation, which are common in older adults with MCI. The guiding principles of CST were adapted to create 15 fundamental principles of individual CST, including mental stimulation, reminiscence, learning, and communication stimulation, using a person-centered approach [12,13].

The study uses an RCT design with experimental, active comparator, and control groups. The primary objectives are to evaluate the effects of the computerized multicomponent cognitive stimulation program on cognitive functions in older adults with MCI and to explore the changes in the EEG and ERP components following the interventions. The study’s structured design, preregistration, and adherence to ethical standards ensure a robust methodology.

The cognitive domains targeted for improvement encompass learning and memory, language, visuospatial abilities, executive functions, psychomotor skills, and emotions. The study integrates various neuropsychological tasks to assess these domains, evaluating the intervention’s impact comprehensively. The expected improvements in these domains align with the program’s focus on CST principles and MSI, which have shown promise in enhancing both cognitive functions and quality of life. It is crucial to have a tablet-based intervention that boosts self-confidence and reduces depression and anxiety. This can involve personalized training, support groups, and skill-building activities to manage cognitive stimulation. For example, previous research has demonstrated that the feasibility and acceptability of the CCS program and computerized cognitive engagement programs were efficient and acceptable, allowing patients with MCI to improve in several aspects of cognitive and psychosocial functioning [16,17]. Computerized technology is cost-effective, and simple to execute, requiring little human and financial resources [18]. In addition, implementing this intervention in middle-income countries may face challenges like setup costs and limited digital infrastructure. However, these barriers can be mitigated through local adaptations and partnerships with community organizations that support technology access and training, making tablet-based programs more applicable across diverse socioeconomic settings [67]. Furthermore, CCS is being used more widely as a standard approach for delivering nonpharmacological interventions to older adults, but determining the effectiveness of nonpharmacological therapies can be challenging [68]. They lack an appraisal of brain activities and a neuropsychological evaluation that correlates with the specific cognitive functions in people with MCI.

The study’s use of EEG and ERP measures to examine neurobiological correlations of MCI adds welcome depth to the investigation. The alterations in brain activity patterns and the ERP components associated with MCI and cognitive impairment contribute to understanding the underlying mechanisms of the condition and identifying potential biomarkers for assessing and monitoring the relevance of the study to treatment. For instance, previous research shows that the EEG of resting-state conditions and a simple cognitive task are important as likely biomarkers for discriminating between healthy, MCI, and Alzheimer groups [69]. It is consistent with Fauzan and Amran [21], finding that when the EEG resting-state measure of the MCI group was compared with typical healthy aging, the theta and alpha increases were more prevalent, showing symptoms of cognitive impairment in MCI, and a decrease in the delta is associated with cognitive decline. In patients with MCI, P300 latency was delayed significantly [26]. P300 latency was related significantly to disease severity in people with probable Alzheimer disease. P300 latency had the highest correlation coefficients with cognitive measurement [23,24]. Furthermore, the N400 component could be connected to verbal memory and learning. The N400 amplitude correlates negatively with semantic expectancy and is inversely proportional to the semantic processing load. P600 has also been linked to memory encoding and retrieval processes [25].

As a result, EEG is a valuable technology for collecting biomedical data from participants pre- and posttest to compare the difference in brain waves after participating in the experiments. However, the limitations of this research are the research design and instrument. The planned follow-up does not take into account that the study may not provide information about the long-term benefits and hazards of medical interventions. Furthermore, EEG data will be collected using the Emotiv Flex which is not capable of native hardware event-marking that can lead to an effect on time-lock stimuli to EEG data [43].

### Limitations and Future Implications

The limitations of this study include its short-term focus, which may capture primarily immediate outcomes while potentially overlooking long-term effectiveness. In addition, the results may have limited generalizability, as they are based on a general population sample and may not extend to specific MCI subtypes. The predominance of female or male participants further limits generalizability, highlighting the need for larger studies to explore potential sex-related variations in response.

Further implications will contribute to knowledge, including developing a novel tablet- and group-based MCST program that combines CST with MSI to improve cognitive functions in older adults with MCI. Using EEG and ERP markers to measure cognitive functions and MSI will provide an assessment of the intervention’s effectiveness. The EEG-recorded results will give a greater understanding of participants’ tablet- and group-based therapeutic data, allowing for the identification of particular mechanisms, such as changes in cognition and emotion, that were found to improve in the pilot study.

### Conclusions

To summarize, the pilot study on older adults with MCI in Thailand revealed significant improvements in cognitive function and emotions following a tablet- and group-based MCST program. The intervention, conducted over 10 weeks for 45 minutes per session, notably enhanced global cognitive function while reducing depression and anxiety levels. These findings corroborate previous research on CCS among patients with MCI. However, contrary to expectations, the MCST program did not yield significant improvements in participants’ quality of life, likely influenced by their awareness of diagnosis and dissatisfaction with cognition and physical well-being. Nonetheless, the study underscores the importance of cognitive interventions and highlights the need for further exploration into specific cognitive domains among older adults with MCI. Further research employing neuropsychological assessments and EEG may provide deeper insights into the mechanisms underlying cognitive performance enhancement in this population.

## Appendix

Multimedia Appendix 1 CONSORT-EHEALTH V1.6 checklist.

## Abbreviations

ANCOVA: one-way analysis of covariance
CCS: computerized cognitive stimulation
CONSORT: Consolidated Standards of Reporting Trials
CST: cognitive stimulation therapy
EEG: electroencephalography
ERP: an event-related potential
JASP: Jeffreys’s Amazing Statistics Program
MCI: mild cognitive impairment
MCST: multicomponent cognitive stimulation therapy
MoCA-T: Thai version of the Montreal Cognitive Assessment
MSI: multisensory integration
OPQoL: Older People’s Quality of Life Questionnaire
RCT: randomized controlled trial
STAI: State-Trait Anxiety Inventory
TGDS: Thai Geriatric Depression Scale

## Data Availability

The datasets generated and analyzed during the current study are available from the corresponding author upon reasonable request.
