# Supplementary material for: Tablet- and Group-Based Multicomponent Cognitive Stimulation for Older Adults With Mild Cognitive Impairment: Single-Group Pilot Study and Protocol for Randomized Controlled Trial
Source: JMIR Res Protoc. 2025 Feb 21;14:e64465. doi: 10.2196/64465 (PMC11890134; doi:10.2196/64465)
Supplement: Multimedia Appendix 1 [file resprot_v14i1e64465_app1.pdf]

CONSORT-EHEALTH (V 1.6.1) - Submission/Publication Form

The CONSORT-EHEALTH checklist is intended for authors of randomized trials evaluating web-based and Internet-based applications/interventions, including mobile interventions, electronic games (incl multiplayer games), social media, certain telehealth applications, and other interactive and/or networked. electronic applications. Some of the items (eg all subitems under item 5 - description of the intervention) may also be applicable for other study designs.

The goal of the CONSORT EHEALTH checklist and guideline is to be
a) a guide for reporting for authors of RCTs,
b) to form a basis for appraisal of an ehealth trial (in terms of validity)

CONSORT-EHEALTH items/subitems are MANDATORY reporting Items for studies published in the Journal of Medical Internet Research and other journals / scientific societies endorsing the checklist.

Items numbered 1., 2., 3., 4a., 4b etc are original CONSORT or CONSORT-NPT (non-pharmacologic treatment) items.
Items with Roman numerals (i., ii, iii, iv etc.) are CONSORT-EHEALTH extensions/clarifications.

As the CONSORT-EHEALTH checklist is still considered in a formative stage, we would ask that you also RATE ON A SCALE OF 1-5 how important/useful you feel each item is FOR THE PURPOSE OF THE CHECKLIST and reporting guideline (optional).

Mandatory reporting items are marked with a red *.
In the textboxes, either copy & paste the relevant sections from your manuscript into this form - please include any quotes from your manuscript in QUOTATION MARKS,
or answer directly by providing additional information not in the manuscript, or elaborating on why the item was not relevant for this study.

YOUR ANSWERS WILL BE PUBLISHED AS A SUPPLEMENTARY FILE TO YOUR PUBLICATION IN JMIR AND ARE CONSIDERED PART OF YOUR PUBLICATION (IF ACCEPTED).
Please fill in these questions diligently. Information will not be copyedited, so please use proper spelling and grammar, use correct capitalization, and avoid abbreviations.

DO NOT FORGET TO SAVE AS PDF _AND_ CLICK THE SUBMIT BUTTON SO YOUR ANSWERS ARE IN OUR DATABASE !!!

Citation Suggestion (if you append the pdf as Appendix we suggest to cite this paper in the caption):
Eysenbach G, CONSORT-EHEALTH Group
CONSORT-EHEALTH: Improving and Standardizing Evaluation Reports of Web-based and Mobile Health Interventions
J Med Internet Res 2011 ;13(4):e126
URL: http://www.jmir.org/2011/4/e126/
doi: 10.2196/jmir.1923
PMID: 22209829

khanitin.j@gmail.com switch account

Not shared

บันทึกฉบับร่างแล้ว

* Indicate that this is a required question.

Your name *

First Last

Khanitin Jornkokgoud

Primary Affiliation (short), City, Country *

University of Toronto, Toronto, Canada

College of Research Methodology and Cognitiv

Your e-mail address *

abc@gmail.com

khanitin.j@gmail.com

Title of your manuscript *

Provide the (draft) title of your manuscript.

Tablet- and Group-based Multicomponent Cognitive Stimulation for Older Adults with Mild Cognitive Impairment: A Single-group Pilot Study and Protocol for a Randomized Controlled Trial

Name of your App/Software/Intervention *

If there is a short and a long/alternate name, write the short name first and add the long name in brackets.

Multicomponent Cognitive Stimulation Therapy

Evaluated Version (if any)

e.g. "V1", "Release 2017-03-01", "Version 2.0.27913"

Your answer

Language(s) *

What language is the intervention/app in? If multiple languages are available, separate by comma (eg "English, French")

Thai Language

URL of your Intervention Website or App

eg a direct link to the mobile app on app in appstore (itunes, Google Play), or URL of the website. If the intervention is a DVD or hardware, you can also link to an Amazon page.

https://manual.cmcs.app/

URL of an image/screenshot (optional)

https://manual.cmcs.app/session-4/section-1

Accessibility *

Can an enduser access the intervention presently?

- ○ Access is free and open
- ◉ access only for special usergroups, not open
- ○ access is open to everyone, but requires payment/subscription/in-app purchases
- ○ app/intervention no longer accessible
- ○ other:

Primary Medical Indication/Disease/Condition *

eg "Stress", "Diabetes", or define the target group in brackets after the condition, eg "Autism (Parents of children with)", "Alzheimers (Informal Caregivers of)"

Mild Cognitive Impairment

Primary Outcomes measured in trial *

comma-separated list of primary outcomes reported in the trial

Learning and memory, executive function, lang

Secondary/other outcomes

Are there any other outcomes the intervention is expected to affect?

A statistically significant increase (P<.05) in the quality of life (OPQoL-Brief score) is expected.

Recommended "Dose" *

What do the instructions for users say on how often the app should be used?

- ○ Approximately Daily
- ◉ Approximately Weekly
- ○ Approximately Monthly
- ○ Approximately Yearly
- ○ "as needed"
- ○ other:

Approx. Percentage of Users (starters) still using the app as recommended after 3 months *

- ○ unknown / not evaluated
- ○ 0-10%
- ○ 11-20%
- ○ 21-30%
- ○ 31-40%
- ◉ 41-50%
- ○ 51-60%
- ○ 61-70%
- ○ 71%-80%
- ○ 81-90%
- ○ 91-100%
- ○ other:

Overall, was the app/intervention effective? *

- ○ yes: all primary outcomes were significantly better in intervention group vs control
- ◉ partly: SOME primary outcomes were significantly better in intervention group vs control
- ○ no statistically significant difference between control and intervention
- ○ potentially harmful: control was significantly better than intervention in one or more outcomes
- ○ inconclusive: more research is needed
- ○ other:

Article Preparation Status/Stage *

At which stage in your article preparation are you currently (at the time you fill in this form)

- ◉ not submitted yet - in early draft status
- ○ not submitted yet - in late draft status, just before submission
- ○ submitted to a journal but not reviewed yet
- ○ submitted to a journal and after receiving initial reviewer comments
- ○ submitted to a journal and accepted, but not published yet
- ○ published
- ○ other:

Journal *

If you already know where you will submit this paper (or if it is already submitted), please provide the journal name (if it is not JMIR, provide the journal name under "other")

- ◉ not submitted yet / unclear where I will submit this
- ○ Journal of Medical Internet Research (JMIR)
- ○ JMIR mHealth and UHealth
- ○ JMIR Serious Games
- ○ JMIR Mental Health
- ○ JMIR Public Health
- ○ JMIR Formative Research
- ○ Other JMIR sister journal
- ○ other:

Is this a full powered effectiveness trial or a pilot/feasibility trial? *

- (selected) Pilot/feasibility
- Fully powered

Manuscript tracking number *

If this is a JMIR submission, please provide the manuscript tracking number under "other" (The ms tracking number can be found in the submission acknowledgment email, or when you login as author in JMIR. If the paper is already published in JMIR, then the ms tracking number is the four-digit number at the end of the DOI, to be found at the bottom of each published article in JMIR)

- no ms number (yet) / not (yet) submitted to / published in JMIR
- (selected) other: 64465

TITLE AND ABSTRACT

1a) TITLE: Identification as a randomized trial in the title

1a) Does your paper address CONSORT item 1a? *

Ie does the title contain the phrase "Randomized Controlled Trial"? (if not, explain the reason under "other")

- (selected) yes
- other:

1a-i) Identify the mode of delivery in the title

Identify the mode of delivery. Preferably use “web-based” and/or “mobile” and/or “electronic game” in the title. Avoid ambiguous terms like “online”, “virtual”, “interactive”. Use “Internet-based” only if Intervention includes non-web-based Internet components (eg email), use “computer-based” or “electronic” only if offline products are used. Use “virtual” only in the context of “virtual reality” (3-D worlds). Use “online” only in the context of “online support groups”. Complement or substitute product names with broader terms for the class of products (such as “mobile” or “smart phone” instead of “iphone”), especially if the application runs on different platforms.

	1	2	3	4	5	
Subitem not at all important	○	○	○	●	○	Essential

Clear Selection

Does your paper address subitem 1a-i? *

Copy and paste relevant sections from manuscript title (include quotes in quotation marks "like this" to indicate direct quotes from your manuscript), or elaborate on this item by providing additional information not in the ms, or briefly explain why the item is not applicable /relevant for your study

"Tablet and Group-based Multicomponent Cognitive Stimulation"

1a-ii) Non-web-based components or important co-interventions in title

Mention non-web-based components or important co-interventions in title, if any (eg, “with telephone support”).

	1	2	3	4	5	
Subitem not at all important	○	●	○	○	○	Essential

Clear Selection

Does your paper address subitem 1a-ii?

Copy and paste relevant sections from manuscript title (include quotes in quotation marks "like this" to indicate direct quotes from your manuscript), or elaborate on this item by providing additional information not in the ms, or briefly explain why the item is not applicable /relevant for your study

"Tablet and Group-based Multicomponent Cognitive Stimulation"

1a-iii) Primary condition or target group in the title

Mention primary condition or target group in the title, if any (eg, “for children with Type I Diabetes”) Example: A Web-based and Mobile Intervention with Telephone Support for Children with Type I Diabetes: Randomized Controlled Trial

	1	2	3	4	5	
Subitem not at all important	○	○	○	○	●	Essential

Clear Selection

Does your paper address subitem 1a-iii? *

Copy and paste relevant sections from manuscript title (include quotes in quotation marks "like this" to indicate direct quotes from your manuscript), or elaborate on this item by providing additional information not in the ms, or briefly explain why the item is not applicable /relevant for your study

"for Older Adults with Mild Cognitive Impairment"

1b) ABSTRACT: Structured summary of trial design, methods, results, and conclusions

NPT extension: Description of experimental treatment, comparator, care providers, centers, and blinding status.

1b-i) Key features/functionalities/components of the intervention and comparator in the METHODS section of the ABSTRACT

Mention key features/functionalities/components of the intervention and comparator in the abstract. If possible, also mention theories and principles used for designing the site. Keep in mind the needs of systematic reviewers and indexers by including important synonyms. (Note: Only report in the abstract what the main paper is reporting. If this information is missing from the main body of text, consider adding it)

	1	2	3	4	5	
Subitem not at all important	○	○	○	○	◉	Essential

Clear Selection

Does your paper address subitem 1b-i? *

Copy and paste relevant sections from the manuscript abstract (include quotes in quotation marks "like this" to indicate direct quotes from your manuscript), or elaborate on this item by providing additional information not in the ms, or briefly explain why the item is not applicable/relevant for your study

"compare tablet-based MCST, tablet-based CST, and control groups"

1b-ii) Level of human involvement in the METHODS section of the ABSTRACT

Clarify the level of human involvement in the abstract, eg, use phrases like “fully automated” vs. “therapist/nurse/care provider/physician-assisted” (mention number and expertise of providers involved, if any). (Note: Only report in the abstract what the main paper is reporting. If this information is missing from the main body of text, consider adding it)

	1	2	3	4	5	
Subitem not at all important	○	○	○	○	◉	Essential

Clear Selection

Does your paper address subitem 1b-ii?

Copy and paste relevant sections from the manuscript abstract (include quotes in quotation marks "like this" to indicate direct quotes from your manuscript), or elaborate on this item by providing additional information not in the ms, or briefly explain why the item is not applicable/relevant for your study

"Intervention groups will be facilitated by trained therapists, nurses, or psychologists. "

1b-iii) Open vs. closed, web-based (self-assessment) vs. face-to-face assessments in the METHODS section of the ABSTRACT

Mention how participants were recruited (online vs. offline), eg, from an open access website or from a clinic or a closed online user group (closed usergroup trial), and clarify if this was a purely web-based trial, or there were face-to-face components (as part of the intervention or for assessment). Clearly say if outcomes were self-assessed through questionnaires (as common in web-based trials). Note: In traditional offline trials, an open trial (open-label trial) is a type of clinical trial in which both the researchers and participants know which treatment is being administered. To avoid confusion, use “blinded” or “unblinded” to indicated the level of blinding instead of “open”, as “open” in web-based trials usually refers to “open access” (ie participants can self-enrol). (Note: Only report in the abstract what the main paper is reporting. If this information is missing from the main body of text, consider adding it)

	1	2	3	4	5	
Subitem not at all important	○	○	○	○	◉	Essential

Clear Selection

Does your paper address subitem 1b-iii?

Copy and paste relevant sections from the manuscript abstract (include quotes in quotation marks "like this" to indicate direct quotes from your manuscript), or elaborate on this item by providing additional information not in the ms, or briefly explain why the item is not applicable/relevant for your study

"All participants will be recruited through closed online user groups."

1b-iv) RESULTS section in abstract must contain use data

Report number of participants enrolled/assessed in each group, the use/uptake of the intervention (eg, attrition/adherence metrics, use over time, number of logins etc.), in addition to primary/secondary outcomes. (Note: Only report in the abstract what the main paper is reporting. If this information is missing from the main body of text, consider adding it)

	1	2	3	4	5	
Subitem not at all important	◉	○	○	○	○	Essential

Clear Selection

Does your paper address subitem 1b-iv?

Copy and paste relevant sections from the manuscript abstract (include quotes in quotation marks "like this" to indicate direct quotes from your manuscript), or elaborate on this item by providing additional information not in the ms, or briefly explain why the item is not applicable/relevant for your study

The study is a protocol for RCT study

1b-v) CONCLUSIONS/DISCUSSION in abstract for negative trials

Conclusions/Discussions in abstract for negative trials: Discuss the primary outcome - if the trial is negative (primary outcome not changed), and the intervention was not used, discuss whether negative results are attributable to lack of uptake and discuss reasons. (Note: Only report in the abstract what the main paper is reporting. If this information is missing from the main body of text, consider adding it)

	1	2	3	4	5	
Subitem not at all important	○	○	○	◉	○	Essential

Clear Selection

Does your paper address subitem 1b-v?

Copy and paste relevant sections from the manuscript abstract (include quotes in quotation marks "like this" to indicate direct quotes from your manuscript), or elaborate on this item by providing additional information not in the ms, or briefly explain why the item is not applicable/relevant for your study

"It will provide deeper insight into participants' neuropsychological data, thus identifying specific processes underlying physiologically measured positive outcomes."

INTRODUCTION

2a) In INTRODUCTION: Scientific background and explanation of rationale

2a-i) Problem and the type of system/solution

Describe the problem and the type of system/solution that is object of the study: intended as stand-alone intervention vs. incorporated in broader health care program? Intended for a particular patient population? Goals of the intervention, eg, being more cost-effective to other interventions, replace or complement other solutions? (Note: Details about the intervention are provided in "Methods" under 5)

	1	2	3	4	5	
Subitem not at all important	○	○	○	○	◉	Essential

Clear Selection

Does your paper address subitem 2a-i? *

Copy and paste relevant sections from the manuscript (include quotes in quotation marks "like this" to indicate direct quotes from your manuscript), or elaborate on this item by providing additional information not in the ms, or briefly explain why the item is not applicable /relevant for your study

"In particular, tablet-based CST should also be further examined in middle-income or developing countries since the expected findings will close the gaps and expand the generalizability of the theories and therapeutic technologies."

2a-ii) Scientific background, rationale: What is known about the (type of) system

Scientific background, rationale: What is known about the (type of) system that is the object of the study (be sure to discuss the use of similar systems for other conditions/diagnoses, if appropiate), motivation for the study, ie what are the reasons for and what is the context for this specific study, from which stakeholder viewpoint is the study performed, potential impact of findings [2]. Briefly justify the choice of the comparator.

	1	2	3	4	5	
Subitem not at all important	○	○	○	●	○	Essential

Clear Selection

Does your paper address subitem 2a-ii? *

Copy and paste relevant sections from the manuscript (include quotes in quotation marks "like this" to indicate direct quotes from your manuscript), or elaborate on this item by providing additional information not in the ms, or briefly explain why the item is not applicable /relevant for your study

"Previous research suggested that the effectiveness of tablet-based CST on cognition, emotion or psychosocial outcomes should be investigated"

2b) In INTRODUCTION: Specific objectives or hypotheses

Does your paper address CONSORT subitem 2b? *

Copy and paste relevant sections from the manuscript (include quotes in quotation marks "like this" to indicate direct quotes from your manuscript), or elaborate on this item by providing additional information not in the ms, or briefly explain why the item is not applicable /relevant for your study

"The following study aims to assess how a tablet-based MCST program, with or without multisensory integration (MSI), affects cognitive functions, emotions, and quality of life in older adults with MCI, comparing experimental and control groups designed as parallel groups. Changes in EEG and ERPs will be examined pre- and post-intervention. The cognitive domains targeted as primary outcomes for improvement include learning and memory, language, visuospatial abilities, executive functions, and psychomotor skills as well as emotions. Specific brain locations are associated with these domains, and the study aims to enhance the functioning of these regions to counteract the cognitive decline observed in MCI. The protocol study hypothesizes that the intervention will improve cognitive functions, emotions, and behaviors in older adults with MCI, outperforming an active comparator and control group. Expected EEG and ERP changes may reveal brain activity patterns tied to cognitive improvements, focusing on frequency bands and ERPs."

METHODS

3a) Description of trial design (such as parallel, factorial) including allocation ratio

Does your paper address CONSORT subitem 3a? *

Copy and paste relevant sections from the manuscript (include quotes in quotation marks "like this" to indicate direct quotes from your manuscript), or elaborate on this item by providing additional information not in the ms, or briefly explain why the item is not applicable /relevant for your study

"This research uses a randomized controlled trial (RCT) design with an experimental group, an active comparator group, and a control group. Participants are randomly assigned to one of the three study conditions. Each participant is randomly allocated 1:1:1 to an intervention or control condition. The study is designed as a randomized, controlled trial with three parallel groups. Randomization will be performed by Random Allocation Software."

3b) Important changes to methods after trial commencement (such as eligibility criteria), with reasons

Does your paper address CONSORT subitem 3b? *

Copy and paste relevant sections from the manuscript (include quotes in quotation marks "like this" to indicate direct quotes from your manuscript), or elaborate on this item by providing additional information not in the ms, or briefly explain why the item is not applicable /relevant for your study

"Inclusion Criteria

Participants eligible for inclusion must meet the following criteria: (1) Language Proficiency: Participants must be native speakers of the Thai language [32] (2) Age Range: The study focuses on individuals aged 60 years or older, (3) Objective Memory Performance: Inclusion is based on objective memory impairment assessments [32] using well-established evaluation tools, including the Thai version of MoCA and CDR[36], (4) Subjective Memory Complaints [32], (5) Handedness: Participants must be right-handed, as confirmed by the Edinburgh Handedness Inventory, (6) Color Vision: Normal color vision is a requirement, evaluated using the Ishihara Plate Test, (7) Health Status: Individuals with no chronic illnesses, deafness, blindness, or an diagnosed neurological disorder are eligible to participate, (8) Cognitive Status: Eligible participants should not exhibit signs of dementia [32], (9) Reading Ability: Prospective participants are expected to be capable of reading and comprehending written material. (10) Computer Proficiency: Participants should be able to use computer devices effectively for the study activities.

Exclusion Criteria

The following criteria will lead to exclusion from participation: (1) A Diagnosis of Dementia: Individuals diagnosed with dementia, irrespective of severity, will not be included in the study. (2) Systemic Illness: Participants with concurrent systemic illnesses that could potentially confound the study outcomes will be excluded. (3) Refusal to Participate: Individuals who decline to participate or express their unwillingness to engage in the study will not be considered for inclusion."

3b-i) Bug fixes, Downtimes, Content Changes

Bug fixes, Downtimes, Content Changes: ehealth systems are often dynamic systems. A description of changes to methods therefore also includes important changes made on the intervention or comparator during the trial (eg, major bug fixes or changes in the functionality or content) (5-iii) and other “unexpected events” that may have influenced study design such as staff changes, system failures/downtimes, etc. [2].

	1	2	3	4	5	
Subitem not at all important	(●)	○	○	○	○	Essential

Clear Selection

Does your paper address subitem 3b-i?

Copy and paste relevant sections from the manuscript (include quotes in quotation marks "like this" to indicate direct quotes from your manuscript), or elaborate on this item by providing additional information not in the ms, or briefly explain why the item is not applicable /relevant for your study

The current state of the protocol study is not focused on system or software

4a) Eligibility criteria for participants

Does your paper address CONSORT subitem 4a? *

Copy and paste relevant sections from the manuscript (include quotes in quotation marks "like this" to indicate direct quotes from your manuscript), or elaborate on this item by providing additional information not in the ms, or briefly explain why the item is not applicable /relevant for your study

"Inclusion Criteria

Participants eligible for inclusion must meet the following criteria: (1) Language Proficiency: Participants must be native speakers of the Thai language [32] (2) Age Range: The study focuses on individuals aged 60 years or older, (3) Objective Memory Performance: Inclusion is based on objective memory impairment assessments [32] using well-established evaluation tools, including the Thai version of MoCA and CDR[36], (4) Subjective Memory Complaints [32], (5) Handedness: Participants must be right-handed, as confirmed by the Edinburgh Handedness Inventory, (6) Color Vision: Normal color vision is a requirement, evaluated using the Ishihara Plate Test, (7) Health Status: Individuals with no chronic illnesses, deafness, blindness, or an diagnosed neurological disorder are eligible to participate, (8) Cognitive Status: Eligible participants should not exhibit signs of dementia [32], (9) Reading Ability: Prospective participants are expected to be capable of reading and comprehending written material. (10) Computer Proficiency: Participants should be able to use computer devices effectively for the study activities.

Exclusion Criteria

The following criteria will lead to exclusion from participation: (1) A Diagnosis of Dementia: Individuals diagnosed with dementia, irrespective of severity, will not be included in the study. (2) Systemic Illness: Participants with concurrent systemic illnesses that could potentially confound the study outcomes will be excluded. (3) Refusal to Participate: Individuals who decline to participate or express their unwillingness to engage in the study will not be considered for inclusion."

4a-i) Computer / Internet literacy

Computer / Internet literacy is often an implicit “de facto” eligibility criterion - this should be explicitly clarified.

	1	2	3	4	5	
Subitem not at all important	(•)	( )	( )	( )	( )	Essential

Clear Selection

Does your paper address subitem 4a-i?

Copy and paste relevant sections from the manuscript (include quotes in quotation marks "like this" to indicate direct quotes from your manuscript), or elaborate on this item by providing additional information not in the ms, or briefly explain why the item is not applicable /relevant for your study

We have the first session, how to use the application, to train participants.

4a-ii) Open vs. closed, web-based vs. face-to-face assessments:

Open vs. closed, web-based vs. face-to-face assessments: Mention how participants were recruited (online vs. offline), eg, from an open access website or from a clinic, and clarify if this was a purely web- based trial, or there were face-to-face components (as part of the intervention or for assessment), ie, to what degree got the study team to know the participant. In online-only trials, clarify if participants were quasi-anonymous and whether having multiple identities was possible or whether technical or logistical measures (eg, cookies, email confirmation, phone calls) were used to detect/prevent these.

	1	2	3	4	5	
Subitem not at all important	○	○	●	○	○	Essential

Clear Selection

Does your paper address subitem 4a-ii? *

Copy and paste relevant sections from the manuscript (include quotes in quotation marks "like this" to indicate direct quotes from your manuscript), or elaborate on this item by providing additional information not in the ms, or briefly explain why the item is not applicable /relevant for your study

"Older adults with MCI will be recruited via communities such as clubs for older adults and elderly schools in the semi-urban area of Chonburi province, Thailand. Announcements regarding the study will be disseminated through radio and social media platforms (Line Messenger, Facebook [Meta], etc). Individuals who express interest will receive information and undergo a screening test. If they meet the inclusion criteria, they will be enrolled in this study."

4a-iii) Information giving during recruitment

Information given during recruitment. Specify how participants were briefed for recruitment and in the informed consent procedures (eg, publish the informed consent documentation as appendix, see also item X26), as this information may have an effect on user self-selection, user expectation and may also bias results. .

	1	2	3	4	5	
Subitem not at all important	○	○	○	○	●	Essential

Clear Selection

Does your paper address subitem 4a-iii?

Copy and paste relevant sections from the manuscript (include quotes in quotation marks "like this" to indicate direct quotes from your manuscript), or elaborate on this item by providing additional information not in the ms, or briefly explain why the item is not applicable /relevant for your study

"Older adults with MCI will be recruited via communities such as clubs for older adults and elderly schools in the semi-urban area of Chonburi province, Thailand. Announcements regarding the study will be disseminated through radio and social media platforms (Line Messenger, Facebook [Meta], etc). Individuals who express interest will receive information and undergo a screening test. If they meet the inclusion criteria, they will be enrolled in this study."

4b) Settings and locations where the data were collected

Does your paper address CONSORT subitem 4b? *

Copy and paste relevant sections from the manuscript (include quotes in quotation marks "like this" to indicate direct quotes from your manuscript), or elaborate on this item by providing additional information not in the ms, or briefly explain why the item is not applicable /relevant for your study

"The study will take place at the Center of Excellence in Cognitive Science (CECoS), which is part of the College of Research Methodology and Cognitive Science (RMCS) at Burapha University."

4b-i) Report if outcomes were (self-)assessed through online questionnaires

Clearly report if outcomes were (self-)assessed through online questionnaires (as common in web-based trials) or otherwise.

	1	2	3	4	5	
Subitem not at all important	◉	○	○	○	○	Essential

Clear Selection

Does your paper address subitem 4b-i? *

Copy and paste relevant sections from the manuscript (include quotes in quotation marks "like this" to indicate direct quotes from your manuscript), or elaborate on this item by providing additional information not in the ms, or briefly explain why the item is not applicable /relevant for your study

We do not have online or web-based questionnaires.

4b-ii) Report how institutional affiliations are displayed

Report how institutional affiliations are displayed to potential participants [on ehealth media], as affiliations with prestigious hospitals or universities may affect volunteer rates, use, and reactions with regards to an intervention.(Not a required item – describe only if this may bias results)

	1	2	3	4	5	
Subitem not at all important	○	○	○	○	◉	Essential

Clear Selection

Does your paper address subitem 4b-ii?

Copy and paste relevant sections from the manuscript (include quotes in quotation marks "like this" to indicate direct quotes from your manuscript), or elaborate on this item by providing additional information not in the ms, or briefly explain why the item is not applicable /relevant for your study

"the College of Research Methodology and Cognitive Science (RMCS) at Burapha [illegible]

5) The interventions for each group with sufficient details to allow replication, including how and when they were actually administered

5-i) Mention names, credential, affiliations of the developers, sponsors, and owners

Mention names, credential, affiliations of the developers, sponsors, and owners [6] (if authors/evaluators are owners or developer of the software, this needs to be declared in a “Conflict of interest” section or mentioned elsewhere in the manuscript) .

	1	2	3	4	5	
Subitem not at all important	○	○	○	○	◉	Essential

Clear Selection

Does your paper address subitem 5-i?

Copy and paste relevant sections from the manuscript (include quotes in quotation marks "like this" to indicate direct quotes from your manuscript), or elaborate on this item by providing additional information not in the ms, or briefly explain why the item is not applicable /relevant for your study

in the section of "Interventions and control group"

5-ii) Describe the history/development process

Describe the history/development process of the application and previous formative evaluations (eg, focus groups, usability testing), as these will have an impact on adoption/use rates and help with interpreting results.

	1	2	3	4	5	
Subitem not at all important	(x)	( )	( )	( )	( )	Essential

Clear Selection

Does your paper address subitem 5-ii?

Copy and paste relevant sections from the manuscript (include quotes in quotation marks "like this" to indicate direct quotes from your manuscript), or elaborate on this item by providing additional information not in the ms, or briefly explain why the item is not applicable /relevant for your study

The current state of the protocol study is not focused on development.

5-iii) Revisions and updating

Revisions and updating. Clearly mention the date and/or version number of the application/intervention (and comparator, if applicable) evaluated, or describe whether the intervention underwent major changes during the evaluation process, or whether the development and/or content was "frozen" during the trial. Describe dynamic components such as news feeds or changing content which may have an impact on the replicability of the intervention (for unexpected events see item 3b).

	1	2	3	4	5	
Subitem not at all important	(x)	( )	( )	( )	( )	Essential

Clear Selection

Does your paper address subitem 5-iii?

Copy and paste relevant sections from the manuscript (include quotes in quotation marks "like this" to indicate direct quotes from your manuscript), or elaborate on this item by providing additional information not in the ms, or briefly explain why the item is not applicable /relevant for your study

The current state of the protocol study is not focused on software development.

5-iv) Quality assurance methods

Provide information on quality assurance methods to ensure accuracy and quality of information provided [1], if applicable.

	1	2	3	4	5	
Subitem not at all important	◉	○	○	○	○	Essential

Clear Selection

Does your paper address subitem 5-iv?

Copy and paste relevant sections from the manuscript (include quotes in quotation marks "like this" to indicate direct quotes from your manuscript), or elaborate on this item by providing additional information not in the ms, or briefly explain why the item is not applicable /relevant for your study

The current state of the protocol study is not focused on software development.

5-v) Ensure replicability by publishing the source code, and/or providing screenshots/screen-capture video, and/or providing flowcharts of the algorithms used

Ensure replicability by publishing the source code, and/or providing screenshots/screen-capture video, and/or providing flowcharts of the algorithms used. Replicability (ie, other researchers should in principle be able to replicate the study) is a hallmark of scientific reporting.

	1	2	3	4	5	
Subitem not at all important	○	○	○	○	◉	Essential

Clear Selection

Does your paper address subitem 5-v?

Copy and paste relevant sections from the manuscript (include quotes in quotation marks "like this" to indicate direct quotes from your manuscript), or elaborate on this item by providing additional information not in the ms, or briefly explain why the item is not applicable /relevant for your study

"Figure 2. Screenshots of the CMCS application: screenshots present kinds of foods in different regions of Thailand."

5-vi) Digital preservation

Digital preservation: Provide the URL of the application, but as the intervention is likely to change or disappear over the course of the years; also make sure the intervention is archived (Internet Archive, webcitation.org , and/or publishing the source code or screenshots/videos alongside the article). As pages behind login screens cannot be archived, consider creating demo pages which are accessible without login.

	1	2	3	4	5	
Subitem not at all important	○	○	●	○	○	Essential

Clear Selection

Does your paper address subitem 5-vi?

Copy and paste relevant sections from the manuscript (include quotes in quotation marks "like this" to indicate direct quotes from your manuscript), or elaborate on this item by providing additional information not in the ms, or briefly explain why the item is not applicable /relevant for your study

"Jornkokgoud, K. CMCS Program Manual. 2024 [cited 2024; Available from: https://manual.cmcs.app."

5-vii) Access

Access: Describe how participants accessed the application, in what setting/context, if they had to pay (or were paid) or not, whether they had to be a member of specific group. If known, describe how participants obtained "access to the platform and Internet" [1]. To ensure access for editors/reviewers/readers, consider to provide a "backdoor" login account or demo mode for reviewers/readers to explore the application (also important for archiving purposes, see vi).

	1	2	3	4	5	
Subitem not at all important	●	○	○	○	○	Essential

Clear Selection

Does your paper address subitem 5-vii? *

Copy and paste relevant sections from the manuscript (include quotes in quotation marks "like this" to indicate direct quotes from your manuscript), or elaborate on this item by providing additional information not in the ms, or briefly explain why the item is not applicable /relevant for your study

The current state of the protocol study is focused on the tablet- and group-based intervention.

5-viii) Mode of delivery, features/functionalities/components of the intervention and comparator, and the theoretical framework

Describe mode of delivery, features/functionalities/components of the intervention and comparator, and the theoretical framework [6] used to design them (instructional strategy [1], behavior change techniques, persuasive features, etc., see eg, [7, 8] for terminology). This includes an in-depth description of the content (including where it is coming from and who developed it) [1]," whether [and how] it is tailored to individual circumstances and allows users to track their progress and receive feedback" [6]. This also includes a description of communication delivery channels and – if computer-mediated communication is a component – whether communication was synchronous or asynchronous [6]. It also includes information on presentation strategies [1], including page design principles, average amount of text on pages, presence of hyperlinks to other resources, etc. [1].

	1	2	3	4	5	
Subitem not at all important	○	○	◉	○	○	Essential

Clear Selection

Does your paper address subitem 5-viii? *

Copy and paste relevant sections from the manuscript (include quotes in quotation marks "like this" to indicate direct quotes from your manuscript), or elaborate on this item by providing additional information not in the ms, or briefly explain why the item is not applicable /relevant for your study

We were described on the section of introduction and interventions. "Experiment intervention"

5-ix) Describe use parameters

Describe use parameters (eg, intended "doses" and optimal timing for use). Clarify what instructions or recommendations were given to the user, eg, regarding timing, frequency, heaviness of use, if any, or was the intervention used ad libitum.

	1	2	3	4	5	
Subitem not at all important	○	○	○	○	◉	Essential

Clear Selection

Does your paper address subitem 5-ix?

Copy and paste relevant sections from the manuscript (include quotes in quotation marks "like this" to indicate direct quotes from your manuscript), or elaborate on this item by providing additional information not in the ms, or briefly explain why the item is not applicable /relevant for your study

We were described on the section of introduction and interventions. "Experiment intervention"

5-x) Clarify the level of human involvement

Clarify the level of human involvement (care providers or health professionals, also technical assistance) in the e-intervention or as co-intervention (detail number and expertise of professionals involved, if any, as well as “type of assistance offered, the timing and frequency of the support, how it is initiated, and the medium by which the assistance is delivered”. It may be necessary to distinguish between the level of human involvement required for the trial, and the level of human involvement required for a routine application. outside of a RCT setting (discuss under item 21 – generalizability).

	1	2	3	4	5	
Subitem not at all important	○	○	○	◉	○	Essential

Clear Selection

Does your paper address subitem 5-x?

Copy and paste relevant sections from the manuscript (include quotes in quotation marks "like this" to indicate direct quotes from your manuscript), or elaborate on this item by providing additional information not in the ms, or briefly explain why the item is not applicable /relevant for your study

"Intervention groups will be facilitated by trained therapists, nurses, or psychologists. "

5-xi) Report any prompts/reminders used

Report any prompts/reminders used: Clarify if there were prompts (letters, emails, phone calls, SMS) to use the application, what triggered them, frequency etc. It may be necessary to distinguish between the level of prompts/reminders required for the trial, and the level of prompts/reminders for a routine application outside of a RCT setting (discuss under item 21 – generalizability).

	1	2	3	4	5	
Subitem not at all important	◉	○	○	○	○	Essential

Clear Selection

Does your paper address subitem 5-xi? *

Copy and paste relevant sections from the manuscript (include quotes in quotation marks "like this" to indicate direct quotes from your manuscript), or elaborate on this item by providing additional information not in the ms, or briefly explain why the item is not applicable /relevant for your study

We do not have any prompts/reminders used in the application.

5-xii) Describe any co-interventions (incl. training/support)

Describe any co-interventions (incl. training/support): Clearly state any interventions that are provided in addition to the targeted eHealth intervention, as ehealth intervention may not be designed as stand-alone intervention. This includes training sessions and support [1]. It may be necessary to distinguish between the level of training required for the trial, and the level of training for a routine application outside of a RCT setting (discuss under item 21 – generalizability.

	1	2	3	4	5	
Subitem not at all important	(●)	( )	( )	( )	( )	Essential

Clear Selection

Does your paper address subitem 5-xii? *

Copy and paste relevant sections from the manuscript (include quotes in quotation marks "like this" to indicate direct quotes from your manuscript), or elaborate on this item by providing additional information not in the ms, or briefly explain why the item is not applicable /relevant for your study

We do not have co-interventions because we would like to specific study on the

6a) Completely defined pre-specified primary and secondary outcome measures, including how and when they were assessed

Does your paper address CONSORT subitem 6a? *

Copy and paste relevant sections from the manuscript (include quotes in quotation marks "like this" to indicate direct quotes from your manuscript), or elaborate on this item by providing additional information not in the ms, or briefly explain why the item is not applicable /relevant for your study

Yes, showing in the section of "Outcomes Measures"

6a-i) Online questionnaires: describe if they were validated for online use and apply CHERRIES items to describe how the questionnaires were designed/deployed

If were obtained through online questionnaires, describe if they were validated for online use and apply CHERRIES items to describe how the questionnaires were designed/deployed [9].

	1	2	3	4	5	
Subitem not at all important	(●)	( )	( )	( )	( )	Essential

Clear Selection

Does your paper address subitem 6a-i?

Copy and paste relevant sections from manuscript text

We do not have online questionnaires.

6a-ii) Describe whether and how “use” (including intensity of use/dosage) was defined/measured/monitored

Describe whether and how “use” (including intensity of use/dosage) was defined/measured/monitored (logins, logfile analysis, etc.). Use/adoption metrics are important process outcomes that should be reported in any ehealth trial.

	1	2	3	4	5	
Subitem not at all important	(●)	( )	( )	( )	( )	Essential

Clear Selection

Does your paper address subitem 6a-ii?

Copy and paste relevant sections from manuscript text

We required all participants to attend all sessions.

6a-iii) Describe whether, how, and when qualitative feedback from participants was obtained

Describe whether, how, and when qualitative feedback from participants was obtained (eg, through emails, feedback forms, interviews, focus groups).

	1	2	3	4	5	
Subitem not at all important	(●)	( )	( )	( )	( )	Essential

Clear Selection

Does your paper address subitem 6a-iii?

Copy and paste relevant sections from manuscript text

We do not collected the qualitative data from participants.

6b) Any changes to trial outcomes after the trial commenced, with reasons

Does your paper address CONSORT subitem 6b? *

Copy and paste relevant sections from the manuscript (include quotes in quotation marks "like this" to indicate direct quotes from your manuscript), or elaborate on this item by providing additional information not in the ms, or briefly explain why the item is not applicable /relevant for your study

We do not changed anay trail outcomes.

7a) How sample size was determined

NPT: When applicable, details of whether and how the clustering by care provides or centers was addressed

7a-i) Describe whether and how expected attrition was taken into account when calculating the sample size

Describe whether and how expected attrition was taken into account when calculating the sample size.

	1	2	3	4	5	
Subitem not at all important	○	○	○	○	●	Essential

Clear Selection

Does your paper address subitem 7a-i?

Copy and paste relevant sections from manuscript title (include quotes in quotation marks "like this" to indicate direct quotes from your manuscript), or elaborate on this item by providing additional information not in the ms, or briefly explain why the item is not applicable /relevant for your study

"The sample size in the study was estimated using the G*Power program [37]. The required sample size considering an alpha = .05, a Cohen's d = .5 (medium magnitude for the effect size) [17, 38], and power = .95, is 26 or 27 participants in each condition, that is, a total of around 80 participants. Assuming a loss of 15% [17, 38, 39], in the event, the final sample consists of 93 participants, approximately 31 participants in each condition"

7b) When applicable, explanation of any interim analyzes and stopping guidelines

Does your paper address CONSORT subitem 7b? *

Copy and paste relevant sections from the manuscript (include quotes in quotation marks "like this" to indicate direct quotes from your manuscript), or elaborate on this item by providing additional information not in the ms, or briefly explain why the item is not applicable /relevant for your study

"Refusal to Participate: Individuals who decline to participate or express their unwillingness to engage in the study will not be considered for inclusion."

8a) Method used to generate the random allocation sequence
NPT: When applicable, how care providers were allocated to each trial group

Does your paper address CONSORT subitem 8a? *

Copy and paste relevant sections from the manuscript (include quotes in quotation marks "like this" to indicate direct quotes from your manuscript), or elaborate on this item by providing additional information not in the ms, or briefly explain why the item is not applicable /relevant for your study

"Randomization will be performed by Random Allocation Software "

8b) Type of randomisation; details of any restriction (such as blocking and block size)

Does your paper address CONSORT subitem 8b? *

Copy and paste relevant sections from the manuscript (include quotes in quotation marks "like this" to indicate direct quotes from your manuscript), or elaborate on this item by providing additional information not in the ms, or briefly explain why the item is not applicable /relevant for your study

"Randomization will be performed by Random Allocation Software "

9) Mechanism used to implement the random allocation sequence (such as sequentially numbered containers), describing any steps taken to conceal the sequence until interventions were assigned

Does your paper address CONSORT subitem 9? *

Copy and paste relevant sections from the manuscript (include quotes in quotation marks "like this" to indicate direct quotes from your manuscript), or elaborate on this item by providing additional information not in the ms, or briefly explain why the item is not applicable /relevant for your study

"Randomization will be performed by Random Allocation Software "

10) Who generated the random allocation sequence, who enrolled participants, and who assigned participants to interventions

Does your paper address CONSORT subitem 10? *

Copy and paste relevant sections from the manuscript (include quotes in quotation marks "like this" to indicate direct quotes from your manuscript), or elaborate on this item by providing additional information not in the ms, or briefly explain why the item is not applicable /relevant for your study

"Randomization will be performed by Random Allocation Software "

11a) If done, who was blinded after assignment to interventions (for example, participants, care providers, those assessing outcomes) and how

NPT: Whether or not administering co-interventions were blinded to group assignment

11a-i) Specify who was blinded, and who wasn't

Specify who was blinded, and who wasn't. Usually, in web-based trials it is not possible to blind the participants [1, 3] (this should be clearly acknowledged), but it may be possible to blind outcome assessors, those doing data analysis or those administering co-interventions (if any).

	1	2	3	4	5	
Subitem not at all important	○	○	○	◉	○	Essential

Clear Selection

Does your paper address subitem 11a-i? *

Copy and paste relevant sections from the manuscript (include quotes in quotation marks "like this" to indicate direct quotes from your manuscript), or elaborate on this item by providing additional information not in the ms, or briefly explain why the item is not applicable /relevant for your study

Participants will be blinded.

11a-ii) Discuss eg, whether participants knew which intervention was the “intervention of interest” and which one was the “comparator”

Informed consent procedures (4a-ii) can create biases and certain expectations - discuss eg, whether participants knew which intervention was the “intervention of interest” and which one was the “comparator”.

	1	2	3	4	5	
Subitem not at all important	○	○	○	○	◉	Essential

Clear Selection

Does your paper address subitem 11a-ii?

Copy and paste relevant sections from the manuscript (include quotes in quotation marks "like this" to indicate direct quotes from your manuscript), or elaborate on this item by providing additional information not in the ms, or briefly explain why the item is not applicable /relevant for your study

"The study draws upon established principles from CST and MSI techniques. The cognitive stimulation program is designed based on group-based CST and multisensory stimulation approaches. CST has been shown to improve cognitive function and quality of life in individuals with dementia or MCI "

11b) If relevant, description of the similarity of interventions

(this item is usually not relevant for ehealth trials as it refers to similarity of a placebo or sham intervention to a active medication/intervention)

Does your paper address CONSORT subitem 11b? *

Copy and paste relevant sections from the manuscript (include quotes in quotation marks "like this" to indicate direct quotes from your manuscript), or elaborate on this item by providing additional information not in the ms, or briefly explain why the item is not applicable /relevant for your study

"Moreover, the group-based cognitive stimulation program, which involves social support from friends and group activities, is critical for mental health and adherence to health-promoting behaviors. It can reduce feelings of loneliness and isolation, which are common in older adults with MCI. The guiding principles of CST were adapted to create 15 fundamental principles of individual CST, including mental stimulation, reminiscence, learning and communication stimulation, employing a person-centered approach "

12a) Statistical methods used to compare groups for primary and secondary outcomes

NPT: When applicable, details of whether and how the clustering by care providers or centers was addressed

Does your paper address CONSORT subitem 12a? *

Copy and paste relevant sections from the manuscript (include quotes in quotation marks "like this" to indicate direct quotes from your manuscript), or elaborate on this item by providing additional information not in the ms, or briefly explain why the item is not applicable /relevant for your study

"Cognitive function scores of the three groups will be compared using one-way analysis of covariance (ANCOVA) to examine differences in post-test scores of various neuropsychological tests controlling for pre-test scores."

12a-i) Imputation techniques to deal with attrition / missing values

Imputation techniques to deal with attrition / missing values: Not all participants will use the intervention/comparator as intended and attrition is typically high in ehealth trials. Specify how participants who did not use the application or dropped out from the trial were treated in the statistical analysis (a complete case analysis is strongly discouraged, and simple imputation techniques such as LOCF may also be problematic [4]).

	1	2	3	4	5	
Subitem not at all important	○	○	○	◉	○	Essential

Clear Selection

Does your paper address subitem 12a-i? *

Copy and paste relevant sections from the manuscript (include quotes in quotation marks "like this" to indicate direct quotes from your manuscript), or elaborate on this item by providing additional information not in the ms, or briefly explain why the item is not applicable /relevant for your study

"Assuming a loss of 15% [17, 38, 39], in the event, the final sample consists of 93 participants, approximately 31 participants in each condition."

12b) Methods for additional analyses, such as subgroup analyzes and adjusted analyses.

Does your paper address CONSORT subitem 12b? *

Copy and paste relevant sections from the manuscript (include quotes in quotation marks "like this" to indicate direct quotes from your manuscript), or elaborate on this item by providing additional information not in the ms, or briefly explain why the item is not applicable /relevant for your study

No, we do not have additional analyses.

X26) REB/IRB Approval and Ethical Considerations [recommended as subheading under "Methods"] (not a CONSORT item)

X26-i) Comment on ethics committee approval

	1	2	3	4	5	
Subitem not at all important	○	○	○	○	◉	Essential

Clear Selection

Does your paper address subitem X26-i?

Copy and paste relevant sections from the manuscript (include quotes in quotation marks "like this" to indicate direct quotes from your manuscript), or elaborate on this item by providing additional information not in the ms, or briefly explain why the item is not applicable /relevant for your study

"Ethical Considerations
The study was approved by the Burapha University Institutional Review Board (Approval number: IRB4-191/2566; Issue date: August 11, 2023 ; Valid until date: August 11, 2024). It was also registered with the Thai Clinical Trials Registry (dated August 29, 2023; TCTR20230829004). All participants provided informed consent. All data will be anonymized. The participants were provided travel compensation (1,200 Thai Baht per participant)."

x26-ii) Outline informed consent procedures

Outline informed consent procedures eg, if consent was obtained offline or online (how? Checkbox, etc.?), and what information was provided (see 4a-ii). See [6] for some items to be included in informed consent documents.

	1	2	3	4	5	
Subitem not at all important	○	○	○	○	◉	Essential

Clear Selection

Does your paper address subitem X26-ii?

Copy and paste relevant sections from the manuscript (include quotes in quotation marks "like this" to indicate direct quotes from your manuscript), or elaborate on this item by providing additional information not in the ms, or briefly explain why the item is not applicable /relevant for your study

"All participants provided informed consent. All data will be anonymized. "

X26-iii) Safety and security procedures

Safety and security procedures, incl. privacy considerations, and any steps taken to reduce the likelihood or detection of harm (eg, education and training, availability of a hotline)

	1	2	3	4	5	
Subitem not at all important	○	○	○	○	○	Essential

Does your paper address subitem X26-iii?

Copy and paste relevant sections from the manuscript (include quotes in quotation marks "like this" to indicate direct quotes from your manuscript), or elaborate on this item by providing additional information not in the ms, or briefly explain why the item is not applicable /relevant for your study

"All participants provided informed consent. All data will be anonymized. "

RESULTS

13a) For each group, the numbers of participants who were randomly assigned, received intended treatment, and were analyzed for the primary outcome.

NPT: The number of care providers or centers performing the intervention in each group and the number of patients treated by each care provider in each center

Does your paper address CONSORT subitem 13a? *

Copy and paste relevant sections from the manuscript (include quotes in quotation marks "like this" to indicate direct quotes from your manuscript), or elaborate on this item by providing additional information not in the ms, or briefly explain why the item is not applicable /relevant for your study

Yes, "Each participant is randomly allocated 1:1:1 to an intervention or control condition."

13b) For each group, losses and exclusions after randomisation, together with reasons

Does your paper address CONSORT subitem 13b? (NOTE: Preferably, this is shown in a CONSORT flow diagram) *

Copy and paste relevant sections from the manuscript (include quotes in quotation marks "like this" to indicate direct quotes from your manuscript), or elaborate on this item by providing additional information not in the ms, or briefly explain why the item is not applicable /relevant for your study

Yes, "Data collection (T1)- No response)

13b-i) Attrition diagram

Strongly recommended: An attrition diagram (eg, proportion of participants still logging in or using the intervention/comparator in each group plotted over time, similar to a survival curve) or other figures or tables demonstrating usage/dose/engagement.

	1	2	3	4	5	
Subitem not at all important	○	○	○	○	◉	Essential

Clear Selection

Does your paper address subitem 13b-i?

Copy and paste relevant sections from the manuscript or cite the figure number if applicable (include quotes in quotation marks "like this" to indicate direct quotes from your manuscript), or elaborate on this item by providing additional information not in the ms, or briefly explain why the item is not applicable/relevant for your study

Yes, "Data collection (T1)- No response)

14a) Dates defining the periods of recruitment and follow-up

Does your paper address CONSORT subitem 14a? *

Copy and paste relevant sections from the manuscript (include quotes in quotation marks "like this" to indicate direct quotes from your manuscript), or elaborate on this item by providing additional information not in the ms, or briefly explain why the item is not applicable /relevant for your study

Yes, "Follow-Up-Data collection (T1)- No response)

14a-i) Indicate if critical “secular events” fell into the study period

Indicate if critical “secular events” fell into the study period, eg, significant changes in Internet resources available or “changes in computer hardware or Internet delivery resources”

	1	2	3	4	5	
Subitem not at all important	◉	○	○	○	○	Essential

Clear Selection

Does your paper address subitem 14a-i?

Copy and paste relevant sections from the manuscript (include quotes in quotation marks "like this" to indicate direct quotes from your manuscript), or elaborate on this item by providing additional information not in the ms, or briefly explain why the item is not applicable /relevant for your study

No, we do not focused on software development.

14b) Why the trial ended or was stopped (early)

Does your paper address CONSORT subitem 14b? *

Copy and paste relevant sections from the manuscript (include quotes in quotation marks "like this" to indicate direct quotes from your manuscript), or elaborate on this item by providing additional information not in the ms, or briefly explain why the item is not applicable /relevant for your study

Yes, at the analysis section.

15) A table showing baseline demographic and clinical characteristics for each group

NPT: When applicable, a description of care providers (case volume, qualification, expertise, etc.) and centers (volume) in each group

Does your paper address CONSORT subitem 15? *

Copy and paste relevant sections from the manuscript (include quotes in quotation marks "like this" to indicate direct quotes from your manuscript), or elaborate on this item by providing additional information not in the ms, or briefly explain why the item is not applicable /relevant for your study

No, the current state is in data analysis, not reporting.

15-i) Report demographics associated with digital divide issues

In ehealth trials it is particularly important to report demographics associated with digital divide issues, such as age, education, gender, social-economic status, computer/Internet/ehealth literacy of the participants, if known.

	1	2	3	4	5	
Subitem not at all important	(●)	( )	( )	( )	( )	Essential

Clear Selection

Does your paper address subitem 15-i? *

Copy and paste relevant sections from the manuscript (include quotes in quotation marks "like this" to indicate direct quotes from your manuscript), or elaborate on this item by providing additional information not in the ms, or briefly explain why the item is not applicable /relevant for your study

No, the current state is in data analysis, not reporting.

16) For each group, number of participants (denominator) included in each analysis and whether the analysis was by original assigned groups

16-i) Report multiple "denominators" and provide definitions

Report multiple "denominators" and provide definitions: Report N's (and effect sizes) "across a range of study participation [and use] thresholds" [1], eg, N exposed, N consented, N used more than x times, N used more than y weeks, N participants "used" the intervention/comparator at specific pre-defined time points of interest (in absolute and relative numbers per group). Always clearly define "use" of the intervention.

	1	2	3	4	5	
Subitem not at all important	◉	○	○	○	○	Essential

Clear Selection

Does your paper address subitem 16-i? *

Copy and paste relevant sections from the manuscript (include quotes in quotation marks "like this" to indicate direct quotes from your manuscript), or elaborate on this item by providing additional information not in the ms, or briefly explain why the item is not applicable /relevant for your study

No, the current state is in data analysis, not reporting.

16-ii) Primary analysis should be intent-to-treat.

Primary analysis should be intent-to-treat, secondary analyzes could include comparing only "users", with the appropriate caveats that this is no longer a randomized sample (see 18-i).

	1	2	3	4	5	
Subitem not at all important	◉	○	○	○	○	Essential

Clear Selection

Does your paper address subitem 16-ii?

Copy and paste relevant sections from the manuscript (include quotes in quotation marks "like this" to indicate direct quotes from your manuscript), or elaborate on this item by providing additional information not in the ms, or briefly explain why the item is not applicable /relevant for your study

No, the current state is in data analysis, not reporting.

17a) For each primary and secondary outcome, results for each group, and the estimated effect size and its precision (such as 95% confidence interval)

Does your paper address CONSORT subitem 17a? *

Copy and paste relevant sections from the manuscript (include quotes in quotation marks "like this" to indicate direct quotes from your manuscript), or elaborate on this item by providing additional information not in the ms, or briefly explain why the item is not applicable /relevant for your study

No, the current state is in data analysis, not reporting.

17a-i) Presentation of process outcomes such as metrics of use and intensity of use

In addition to primary/secondary (clinical) outcomes, the presentation of process outcomes such as metrics of use and intensity of use (dose, exposure) and their operational definitions is critical. This does not only refer to metrics of attrition (13-b) (often a binary variable), but also to more continuous exposure metrics such as “average session length”. These must be accompanied by a technical description how a metric like a “session” is defined (eg, timeout after idle time) [1] (report under item 6a).

	1	2	3	4	5	
Subitem not at all important	◉	○	○	○	○	Essential

Clear Selection

Does your paper address subitem 17a-i?

Copy and paste relevant sections from the manuscript (include quotes in quotation marks "like this" to indicate direct quotes from your manuscript), or elaborate on this item by providing additional information not in the ms, or briefly explain why the item is not applicable /relevant for your study

No, the current state is in data analysis, not reporting.

17b) For binary outcomes, presentation of both absolute and relative effect sizes is recommended.

Does your paper address CONSORT subitem 17b? *

Copy and paste relevant sections from the manuscript (include quotes in quotation marks "like this" to indicate direct quotes from your manuscript), or elaborate on this item by providing additional information not in the ms, or briefly explain why the item is not applicable /relevant for your study

No, the current state is in data analysis, not reporting. However, we plan to report effect sizes for outcomes.

18) Results of any other analyzes performed, including subgroup analyzes and adjusted analyses, distinguishing pre-specified from exploratory

Does your paper address CONSORT subitem 18? *

Copy and paste relevant sections from the manuscript (include quotes in quotation marks "like this" to indicate direct quotes from your manuscript), or elaborate on this item by providing additional information not in the ms, or briefly explain why the item is not applicable /relevant for your study

No, we do not plan to subgroup analyse.

18-i) Subgroup analysis of comparing only users

A subgroup analysis of comparing only users is not uncommon in ehealth trials, but if done, it must be stressed that this is a self-selected sample and no longer an unbiased sample from a randomized trial (see 16-iii).

	1	2	3	4	5	
Subitem not at all important	◉	○	○	○	○	Essential

Clear Selection

Does your paper address subitem 18-i?

Copy and paste relevant sections from the manuscript (include quotes in quotation marks "like this" to indicate direct quotes from your manuscript), or elaborate on this item by providing additional information not in the ms, or briefly explain why the item is not applicable /relevant for your study

No, we do not plan to subgroup analyse.

19) All important harms or unintended effects in each group

(for specific guidance see CONSORT for harms)

Does your paper address CONSORT subitem 19? *

Copy and paste relevant sections from the manuscript (include quotes in quotation marks "like this" to indicate direct quotes from your manuscript), or elaborate on this item by providing additional information not in the ms, or briefly explain why the item is not applicable /relevant for your study

Yes, we declared to the Burapha University Institutional Review Board.

19-i) Include privacy breaches, technical problems

Include privacy breaches, technical problems. This does not only include physical "harm" to participants, but also incidents such as perceived or real privacy breaches [1], technical problems, and other unexpected/unintended incidents. "Unintended effects" also includes unintended positive effects [2].

	1	2	3	4	5	
Subitem not at all important	○	○	○	○	◉	Essential

Clear Selection

Does your paper address subitem 19-i?

Copy and paste relevant sections from the manuscript (include quotes in quotation marks "like this" to indicate direct quotes from your manuscript), or elaborate on this item by providing additional information not in the ms, or briefly explain why the item is not applicable /relevant for your study

Yes, we declared to the Burapha University Institutional Review Board.

19-ii) Include qualitative feedback from participants or observations from staff/researchers

Include qualitative feedback from participants or observations from staff/researchers, if available, on strengths and shortcomings of the application, especially if they point to unintended/unexpected effects or uses. This includes (if available) reasons for why people did or did not use the application as intended by the developers.

	1	2	3	4	5	
Subitem not at all important	◉	○	○	○	○	Essential

Clear Selection

Does your paper address subitem 19-ii?

Copy and paste relevant sections from the manuscript (include quotes in quotation marks "like this" to indicate direct quotes from your manuscript), or elaborate on this item by providing additional information not in the ms, or briefly explain why the item is not applicable /relevant for your study

No, we do not collecting data on aualitative feedback.

DISCUSSION

22) Interpretation consistent with results, balancing benefits and harms, and considering other relevant evidence

NPT: In addition, take into account the choice of the comparator, lack of or partial blinding, and unequal expertise of care providers or centers in each group

22-i) Restate study questions and summarize the answers suggested by the data, starting with primary outcomes and process outcomes (use)

Restate study questions and summarize the answers suggested by the data, starting with primary outcomes and process outcomes (use).

	1	2	3	4	5	
Subitem not at all important	○	○	○	●	○	Essential

Clear Selection

Does your paper address subitem 22-i? *

Copy and paste relevant sections from the manuscript (include quotes in quotation marks "like this" to indicate direct quotes from your manuscript), or elaborate on this item by providing additional information not in the ms, or briefly explain why the item is not applicable /relevant for your study

Yes, it showed on "Principal Results for the RCT Study" section

22-ii) Highlight unanswered new questions, suggest future research

Highlight unanswered new questions, suggest future research.

	1	2	3	4	5	
Subitem not at all important	○	○	○	●	○	Essential

Clear Selection

Does your paper address subitem 22-ii?

Copy and paste relevant sections from the manuscript (include quotes in quotation marks "like this" to indicate direct quotes from your manuscript), or elaborate on this item by providing additional information not in the ms, or briefly explain why the item is not applicable /relevant for your study

Yes, it presented on "Limitations and Future Implications"

20) Trial limitations, addressing sources of potential bias, imprecision, and, if relevant, multiplicity of analyses

20-i) Typical limitations in ehealth trials

Typical limitations in ehealth trials: Participants in ehealth trials are rarely blinded. Ehealth trials often look at a multiplicity of outcomes, increasing risk for a Type I error. Discuss biases due to non-use of the intervention/usability issues, biases through informed consent procedures, unexpected events.

	1	2	3	4	5	
Subitem not at all important	○	○	○	●	○	Essential

Clear Selection

Does your paper address subitem 20-i? *

Copy and paste relevant sections from the manuscript (include quotes in quotation marks "like this" to indicate direct quotes from your manuscript), or elaborate on this item by providing additional information not in the ms, or briefly explain why the item is not applicable /relevant for your study

Yes, it presented on "Limitations and Future Implications"

21) Generalisability (external validity, applicability) of the trial findings

NPT: External validity of the trial findings according to the intervention, comparators, patients, and care providers or centers involved in the trial

21-i) Generalizability to other populations

Generalizability to other populations: In particular, discuss generalizability to a general Internet population, outside of a RCT setting, and general patient population, including applicability of the study results for other organizations.

	1	2	3	4	5	
Subitem not at all important	○	○	○	●	○	Essential

Clear Selection

Does your paper address subitem 21-i?

Copy and paste relevant sections from the manuscript (include quotes in quotation marks "like this" to indicate direct quotes from your manuscript), or elaborate on this item by providing additional information not in the ms, or briefly explain why the item is not applicable /relevant for your study

Yes, it presented on "Limitations and Future Implications and conclusion"

21-ii) Discuss if there were elements in the RCT that would be different in a routine application setting

Discuss if there were elements in the RCT that would be different in a routine application setting (eg, prompts/reminders, more human involvement, training sessions or other co-interventions) and what impact the omission of these elements could have on use, adoption , or outcomes if the intervention is applied outside of a RCT setting.

	1	2	3	4	5	
Subitem not at all important	(•)	( )	( )	( )	( )	Essential

Clear Selection

Does your paper address subitem 21-ii?

Copy and paste relevant sections from the manuscript (include quotes in quotation marks "like this" to indicate direct quotes from your manuscript), or elaborate on this item by providing additional information not in the ms, or briefly explain why the item is not applicable /relevant for your study

No, we were discuss in term of intervention approach not about training sessions.

OTHER INFORMATION

23) Registration number and name of trial registry

Does your paper address CONSORT subitem 23? *

Copy and paste relevant sections from the manuscript (include quotes in quotation marks "like this" to indicate direct quotes from your manuscript), or elaborate on this item by providing additional information not in the ms, or briefly explain why the item is not applicable /relevant for your study

Yes, Thai Clinical Trials Registry (reference number: TCTR20230829004).

24) Where the full trial protocol can be accessed, if available

Does your paper address CONSORT subitem 24? *

Cite a Multimedia Appendix, other reference, or copy and paste relevant sections from the manuscript (include quotes in quotation marks "like this" to indicate direct quotes from your manuscript), or elaborate on this item by providing additional information not in the ms, or briefly explain why the item is not applicable/relevant for your study

Yes, https://www.thaiclinicaltrials.org/show/TCTR20230829004

25) Sources of funding and other support (such as supply of drugs), role of funders

Does your paper address CONSORT subitem 25? *

Copy and paste relevant sections from the manuscript (include quotes in quotation marks "like this" to indicate direct quotes from your manuscript), or elaborate on this item by providing additional information not in the ms, or briefly explain why the item is not applicable /relevant for your study

Yes, it provided in Thai Clinical Trials Registry and the manuscript

X27) Conflicts of Interest (not a CONSORT item)

X27-i) State the relation of the study team towards the system being evaluated

In addition to the usual declaration of interests (financial or otherwise), also state the relation of the study team towards the system being evaluated, ie, state if the authors/evaluators are distinct from or identical with the developers/sponsors of the intervention.

	1	2	3	4	5	
Subitem not at all important	○	○	○	○	◉	Essential

Clear Selection

Does your paper address subitem X27-i?

Copy and paste relevant sections from the manuscript (include quotes in quotation marks "like this" to indicate direct quotes from your manuscript), or elaborate on this item by providing additional information not in the ms, or briefly explain why the item is not applicable /relevant for your study

"The authors declare no conflicts of interest."

About the CONSORT EHEALTH checklist

As a result of using this checklist, did you make changes in your manuscript? *

- ( ) yes, major changes
- (•) yes, minor changes
- ( ) no

What were the most important changes you made as a result of using this checklist?

We added some sections that relate to the CONSORT checklist.

How much time did you spend on going through the checklist INCLUDING making changes in your manuscript *

We spend more than two weeks.

As a result of using this checklist, do you think your manuscript has improved? *

- (•) yes
- ( ) no
- ( ) other:

Would you like to become involved in the CONSORT EHEALTH group?
This would involve for example becoming involved in participating in a workshop and writing an "Explanation and Elaboration" document

- (•) yes
- ( ) no
- ( ) other:

Clear Selection

Any other comments or questions on CONSORT EHEALTH

The CONSORT-EHEALTH checklist is highly beneficial, as it helps us to present our manuscript with greater clarity and transparency. It ensures that all essential elements are covered, facilitating a more thorough and organized report on our study.

STOP - Save this form as PDF before you click submit
To generate a record that you filled in this form, we recommend to generate a PDF of this page (on a Mac, simply select "print" and then select "print as PDF") before you submit it.

When you submit your (revised) paper to JMIR, please upload the PDF as supplementary file.

Don't worry if some text in the textboxes is cut off, as we still have the complete information in our database. Thank you!

Final step: Click submit !
Click submit so we have your answers in our database!

send Clear Form

Do not submit passwords in Google Forms

This content is not created or endorsed by Google. Report Abuse - Terms of Service - Privacy Policy

Google form
